# Nutritional vulnerability, food insecurity, and unmet supportive care needs in multiple myeloma: a cross-sectional study from China

**DOI:** 10.3389/fnut.2026.1773188

**Published:** 2026-04-08

**Authors:** Jin Zhao, Xiaolian Wen, Li Ma, Xiaojing Guo, Liping Su

**Affiliations:** 1 Department of Hematology, Shanxi Medical University Affiliated Cancer Hospital/Shanxi, Provincial Cancer Hospital/Chinese Academy of Medical Sciences Cancer Hospital Shanxi, Taiyuan, Shanxi, China; 2 Research on Precision Diagnosis and Treatment of Lymphoma in the Hematology Department of Shanxi Provincial Key Laboratory, Taiyuan, Shanxi, China

**Keywords:** food insecurity, multiple myeloma, nutritional vulnerability, quality of life, supportive care, symptom burden

## Abstract

**Background:**

Multiple myeloma patients experience nutritional vulnerability through disease-related factors, treatment toxicity, and systemic inflammation, yet comprehensive assessment integrating validated nutrition screening with health equity dimensions remains limited. This study characterized nutritional vulnerability prevalence, determinants, and supportive-care access barriers among Chinese multiple myeloma patients.

**Methods:**

This single-center, cross-sectional observational study conducted at Shanxi Provincial Key Laboratory, Taiyuan, China (January 2020–December 2024) employed comprehensive assessment including Patient-Generated Subjective Global Assessment Short Form (PG-SGA-SF), Edmonton Symptom Assessment System (ESAS), EORTC QLQ-C30/MY20 quality of life questionnaires, anthropometry, handgrip strength, biochemical markers, food insecurity screening and systematic barriers evaluation. Multivariable logistic regression identified independent predictors of composite nutritional vulnerability; principal component analysis explored multidimensional phenotype structure.

**Results:**

Among 286 patients completing assessment (mean age 59.8 ± 13.0 years; 52.4% male; 49.3% relapsed/refractory), 57.3% (95% CI: 51.6–62.9) had moderate-to-high nutritional risk, including 7.0% with critical vulnerability. Risk increased across treatment phases (50.7% newly diagnosed, 44.7% remission, 63.7% relapsed, 74.0% refractory; p-trend = 0.002), and composite nutritional vulnerability affected 61.2%. High symptom burden (ESAS ≥40) was dominant independent correlate (adjusted OR = 5.03, 95% CI: 3.08–10.05; *p* < 0.001), whereas disease stage and treatment intensity were not independently associated. Despite risk, 61.6% of affected patients had unmet nutritional support needs. Oral nutritional supplement use occurred in 45.1% overall, with only modest variation by risk status (42.6–50.0%; *p* = 0.035). Food insecurity affected 48.3% of patients, demonstrated a strong income gradient (*p* < 0.001), and remained independently associated with vulnerability (adjusted OR = 2.12, 95% CI: 1.39–3.24). Nutritional vulnerability was associated with large quality-of-life deficits in global health, physical, and role functioning (adjusted mean differences 35.9–40.5 points; all *p* < 0.001).

**Conclusion:**

Nutritional vulnerability in multiple myeloma is highly prevalent, symptom-driven, and inadequately addressed despite devastating quality of life impact, with food insecurity and systemic access barriers compounding clinical risk among socioeconomically vulnerable populations.

## 1 Introduction

Multiple myeloma (MM) is an incurable plasma-cell malignancy that imposes a growing burden on health systems through recurrent treatment, cumulative toxicity, and long-term impairment in function and wellbeing (1, 2). Contemporary epidemiologic analyses indicate that MM burden is increasing in many settings, and China is experiencing sustained growth in incidence and disability as population ageing accelerates and survival improves with novel therapeutics (2–4). In a recent national burden analysis using Global Burden of Disease methods, China was estimated to have 18,793 incident MM cases and 13,421 deaths in 2019, with an age-standardized incidence rate of 0.93 per 100,000 and an age-standardized mortality rate of 0.67 per 100,000; the corresponding disability-adjusted life years were estimated at 347.45 thousand with an age-standardized DALY rate of 17.05 per 100,000, underscoring a material and increasing public-health burden extending beyond clinical outcomes alone (1, 2). Notably, insurance-claims–based estimates from urban basic medical insurance have further quantified the expanding prevalence and incidence of MM within China, supporting the contention that health-service demand will continue to rise across the care continuum (4, 5). These trends render supportive-care optimization—including nutritional care, symptom control, and quality-of-life (QoL) preservation—an increasingly important dimension of high-value MM management (6–8).

Cancer-associated malnutrition and related syndromes, including sarcopenia and cachexia, are highly prevalent, clinically consequential, and, importantly, modifiable targets for intervention (9, 10). International expert recommendations and evidence-based guidelines emphasize that nutrition risk should be screened early and repeatedly throughout the disease course, and that assessment should move beyond weight alone to include reduced intake, inflammation, body composition, and physical function (6, 7). Within oncology broadly, malnutrition has been consistently associated with inferior tolerance of systemic therapy, higher complication rates, impaired function, and worse survival, and systematic reviews have reinforced that validated tools such as the Patient-Generated Subjective Global Assessment (PG-SGA) provide prognostically informative stratification across cancer types (7, 11). Moreover, in MM specifically, disease- and treatment-related factors plausibly intensify nutrition vulnerability through anorexia, gastrointestinal toxicity, pain, fatigue, neuropathy, corticosteroid exposure, infection risk, and systemic inflammation, thereby disrupting dietary intake, physical activity, and muscle maintenance across lines of therapy (9, 10, 12, 13). Unlike solid tumors—where acute weight loss and sarcopenia frequently dominate the malnutrition phenotype at diagnosis—MM presents a distinctive nutritional trajectory characterized by early and progressive symptom-mediated intake impairment, inflammation-driven hypoalbuminemia, and cumulative functional decline rather than rapid catabolic wasting; this pattern intensifies across successive lines of therapy as cumulative toxicity and repeated disease progression accrue. Understanding this disease-specific timing and mechanistic profile is therefore essential for designing appropriately targeted nutritional screening and intervention strategies in MM.

Validated nutrition-risk instruments provide a methodological backbone for high-impact clinical nutrition research because they anchor measurement to established constructs and allow comparability across studies (14, 15). The PG-SGA has been widely used in oncology and has shown clinically meaningful associations with outcomes; in a study of newly diagnosed MM, pre-chemotherapy PG-SGA scores were linked to clinical characteristics and overall survival, supporting the conceptual and prognostic relevance of structured nutrition assessment in this disease (12, 16). In parallel, contemporary syntheses have evaluated malnutrition definitions and screening and diagnostic performance frameworks, including comparisons between the Global Leadership Initiative on Malnutrition (GLIM) criteria and PG-SGA, further highlighting the importance of systematic, standardized measurement rather than *ad hoc* indices (14, 17, 18). Nevertheless, while validated tools capture core malnutrition domains, high-impact nutritional supportive-care studies increasingly integrate objective anthropometry, functional markers such as handgrip strength, inflammation-sensitive biomarkers including C-reactive protein, and patient-reported symptom burden, because these complementary measures offer mechanistic granularity and improve clinical interpretability (6, 7, 13).

Symptom burden is a central pathway linking MM, its treatment, and downstream nutritional vulnerability, because symptoms such as nausea, constipation or diarrhea, mucositis, pain, fatigue, neuropathy, anxiety, and depression can reduce oral intake and impair the capacity for meal preparation and physical activity (19–22). The Edmonton Symptom Assessment System (ESAS) has been extensively used in oncology, with well-described feasibility, validity, and responsiveness, and has become a pragmatic standard for capturing multidimensional symptom severity in clinical practice and research (22). Importantly, symptom burden is not only an intermediate factor but also a clinically meaningful outcome itself, making symptom–nutrition interrelationships particularly salient for supportive-care–oriented nutrition journals (19, 23, 24).

Health-related quality of life serves as a critical integrative outcome reflecting the synergistic impacts of nutritional status, symptom burden, and psychosocial stressors, with the European Organisation for Research and Treatment of Cancer (EORTC), QLQ-C30 and the validated Chinese version of the myeloma-specific QLQ-MY20 providing reliable instruments for assessing these domains in Chinese populations (25–28). However, a substantive gap remains as global literature frequently treats malnutrition, symptoms, and QoL as parallel constructs rather than an integrated vulnerability phenotype that incorporates care access, financial toxicity, and socioeconomic constraints (19, 29, 30). Despite the availability of translated tools like the U.S. Household Food Security Survey Module, there is limited integration of food insecurity, financial hardship, and geographic barriers with validated clinical nutrition risk tools in hematologic malignancy research, particularly within the Chinese context, where these factors may independently or synergistically compromise nutritional adequacy and dietitian utilization (27, 31–33).

Within China, several contextual factors plausibly strengthen the rationale for a comprehensive nutritional vulnerability and supportive-care access assessment. First, the rising disease burden and ageing demographics increase the proportion of patients at baseline risk for sarcopenia and functional decline (1, 2, 34). Second, treatment patterns and supportive-care infrastructure vary across settings, and well-documented rural–urban differentials may influence travel time, access to dietitians, affordability of oral nutritional supplements, and continuity of symptom-management services (35, 36). Third, dietary exposures and counselling targets are shaped by local dietary norms and policy guidance; for example, Chinese dietary recommendations are commonly operationalized using gram-based targets for vegetables (300–500 g/day) and fruits (200–350 g/day), which can be incorporated into culturally relevant assessment of dietary adequacy (37). In parallel, Traditional Chinese Medicine use is prevalent and may influence dietary behaviors, beliefs about nutrition, and communication with oncology teams, creating a uniquely relevant domain for China-based nutritional supportive-care research (38–40).

This single-center study in China employs a methodologically rigorous framework to bridge the gap between clinical nutrition and health equity in multiple myeloma care through three primary objectives. First, it aims to establish a multi-dimensional nutritional profile by quantifying the prevalence and severity of nutritional vulnerability, integrating the PG-SGA with objective anthropometry, functional capacity measures like handgrip strength, and biochemical laboratory markers. Second, the study evaluates the impact of nutritional status on patient-reported outcomes, specifically analyzing how nutritional decline correlates with symptom burden via the ESAS and health-related quality of life via the EORTC QLQ-C30 and its myeloma-specific module, while identifying if these risks concentrate within specific treatment phases. Finally, the research seeks to characterize the systemic determinants of supportive-care access by examining how socioeconomic status, geographic location, and food insecurity interact with financial toxicity and limited dietitian utilization to amplify unmet needs among the most clinically and socially vulnerable patients.

## 2 Methods

### 2.1 Study design and setting

This single-center, cross-sectional observational study was conducted at the Research on Precision Diagnosis and Treatment of Lymphoma in the Hematology Department of Shanxi Provincial Key Laboratory, Taiyuan, Shanxi, 030013, China, between January 2020 and December 2024. The study employed a comprehensive assessment framework integrating validated nutrition-risk screening instruments, patient-reported outcome measures, objective anthropometry, functional capacity testing, and biochemical markers to characterize nutritional vulnerability and supportive-care access among patients with multiple myeloma across the disease trajectory.

### 2.2 Study population and sampling

#### 2.2.1 Eligibility criteria and recruitment

Consecutive adult patients (aged ≥18 years) with histologically confirmed multiple myeloma were consecutively enrolled during routine outpatient visits or inpatient admissions across all treatment phases: newly diagnosed (treatment-naïve), in remission (complete/partial response maintained ≥3 months), relapsed (disease progression after previous response), and refractory (failure to achieve minimal response or progression within 60 days of last therapy). Patients required adequate cognitive function to complete self-administered questionnaires independently or with assistance from trained research personnel. Exclusion criteria comprised: (1) concurrent active malignancy other than multiple myeloma; (2) severe psychiatric illness or cognitive impairment precluding informed consent or questionnaire completion; (3) pregnancy or lactation; (4) inability to communicate in Mandarin Chinese; and (5) participation in investigational nutrition intervention trials that could confound baseline nutritional assessment. Because PG-SGA-SF completion required adequate functional and cognitive capacity, patients with Eastern Cooperative Oncology Group (ECOG) performance status ≥2 were under-represented in the complete-case analytic cohort, resulting in complete absence of ECOG ≥2 observations in primary analyses. ECOG was retained in multivariable models for conceptual completeness and comparability with prior literature, but estimates should be interpreted cautiously given the restricted range.

A total of 416 consecutive patients with confirmed multiple myeloma were screened for eligibility between January 2020 and December 2024. Of these, 116 patients (27.9%) were excluded during screening due to ECOG performance status ≥2 (*n* = 88), active concurrent malignancy (*n* = 12), cognitive impairment precluding consent (*n* = 9), inability to communicate in Mandarin (*n* = 4), and participation in investigational nutrition trials (*n* = 3), yielding 300 eligible patients who were enrolled. Food insecurity assessment was completed by all 300 enrolled patients prior to other assessments (denominator *N* = 300 for food insecurity analyses). Of the 300 enrolled patients, 286 (95.3%) completed all required assessments and comprised the primary analytic cohort (denominator *N* = 286 for all other analyses). Fourteen patients (4.7%) were excluded post-enrollment due to incomplete PG-SGA-SF data (*n* = 8), inability to complete ESAS (*n* = 4), or withdrawal of consent (*n* = 2). Food insecurity assessment was completed by all 300 enrolled patients prior to other assessments. Importantly, the eligibility requirement for ECOG 0–1 functional status to permit self-administered questionnaire completion resulted in the exclusion of 116 patients during screening (38.7% of all 416 patients screened). Compared with the analytic cohort (*n* = 286), excluded ECOG ≥2 patients had significantly higher proportions of ISS stage III disease (42.2% vs. 22.4%, *p* < 0.001), relapsed or refractory disease (68.1% vs. 49.3%, *p* = 0.002), and lower median serum albumin (36.2 vs. 40.0 g/L, *p* < 0.001), confirming systematic exclusion of patients with the greatest clinical and likely nutritional severity. All reported prevalence estimates therefore represent conservative lower bounds applicable specifically to functionally preserved multiple myeloma patients (ECOG 0–1).

#### 2.2.2 Disease and treatment classification

Treatment phase was determined through medical record review and oncologist confirmation. Disease staging followed the International Staging System (ISS): ISS stage I (β2-microglobulin <3.5 mg/L and albumin ≥35 g/L), II (neither I nor III), or III (β2-microglobulin ≥5.5 mg/L) (41). Performance status was assessed using the ECOG scale (0–4) (42), theoretically dichotomized as 0–1 versus ≥2; however, minimal representation of ECOG ≥2 patients occurred due to functional capacity requirements for questionnaire completion. Relapsed disease was defined as progression requiring new treatment after achieving minimal response or better; refractory disease as progression on therapy or within 60 days of last treatment, per International Myeloma Working Group consensus definitions (43).

### 2.3 Data collection procedures

Data collection occurred during a single comprehensive assessment visit (approximately 45–60 min) conducted by trained research nurses and a registered dietitian with specialized oncology nutrition certification. Participants completed structured questionnaires in a private clinic setting with research personnel available to clarify items while maintaining standardization. Clinical data were abstracted from electronic medical records using standardized case report forms pilot-tested for completeness and inter-rater reliability. Anthropometric measurements and handgrip strength testing were performed by certified research staff with demonstrated inter-rater reliability (intraclass correlation >0.95 for all continuous measurements). Venous blood samples collected within 14 days of assessment provided laboratory parameters from the hospital’s ISO 15189-accredited central laboratory; when multiple values existed within the 28-day window, the value closest to questionnaire completion was used.

### 2.4 Sociodemographic variables

#### 2.4.1 Individual and household characteristics

Age was recorded in completed years and analyzed continuously (per 10-year increments in regression models) and categorically by decade. Sex was documented as male or female from legal identification. Educational attainment was assessed as: (1) primary school or below (≤6 years); (2) middle school (7–9 years); (3) high school/vocational training (10–12 years); or (4) college/university or above (>12 years), dichotomized as low (≤middle school) versus college or above for analysis. Residence type was classified as urban or rural based on household registration (hukou) status and current dwelling location following Chinese National Bureau of Statistics criteria. Household monthly income was self-reported categorically: <3,000 RMB, 3,000–5,999 RMB, 6,000–9,999 RMB, or ≥10,000 RMB, with categorical collection minimizing social desirability bias and non-response. Low household income was defined as <3,000 RMB/month (approximately <420 USD), corresponding to the lower tertile of national urban disposable income. Health insurance coverage was documented as urban employee basic medical insurance, urban–rural resident basic medical insurance, or no coverage, with notation of supplemental commercial insurance.

### 2.5 Clinical disease and treatment variables

#### 2.5.1 Characteristics of disease and staging

Disease characteristics including ISS stage (I, II, III), cytogenetic risk stratification (standard versus high-risk by fluorescence *in situ* hybridization), and immunoglobulin subtype (IgG, IgA, light chain only, non-secretory) were extracted from diagnostic reports; for patients diagnosed prior to enrollment, the most recent staging evaluation was used. ISS stage was analyzed as three-category ordinal and dichotomized as stage III versus I–II. Cytogenetic risk and immunoglobulin subtype were recorded descriptively but not included in primary multivariable models due to sample size constraints and to maintain model parsimony focused on nutritional and supportive-care determinants.

#### 2.5.2 Treatment history and current status

Treatment history documentation included: date of initial diagnosis, number of prior treatment lines (distinct regimens separated by disease progression, toxicity-driven discontinuation, or planned sequential approaches), current regimen components (proteasome inhibitors, immunomodulatory agents, corticosteroids, alkylating chemotherapy, autologous stem cell transplantation), treatment phase, and disease status (complete response, very good partial response, partial response, stable disease, or progressive disease per International Myeloma Working Group criteria). Treatment line was analyzed continuously and dichotomized as first-line versus ≥2 lines. Relapsed/refractory disease status was a composite binary variable indicating either relapsed or refractory disease per International Myeloma Working Group definitions. Unplanned hospitalization was assessed via self-report with medical record verification, defined as any emergency department visit resulting in admission or unscheduled inpatient stay in the preceding 3 months, excluding planned admissions for scheduled chemotherapy or autologous transplantation, serving as a proxy for acute clinical deterioration, treatment complications, or inadequate outpatient symptom management.

### 2.6 Anthropometric and functional measurements

#### 2.6.1 Body composition indices

Body weight was measured to the nearest 0.1 kg using a calibrated digital scale (SECA 877, Hamburg, Germany; weekly calibration with certified reference weights) with participants wearing light indoor clothing without shoes after voiding. Height was measured to the nearest 0.1 cm using a wall-mounted stadiometer (SECA 217) with participants standing erect, heels together, scapulae and buttocks contacting the vertical backboard, and head in the Frankfort horizontal plane; for participants unable to stand erect due to skeletal complications or pain, self-reported historical height verified through medical records was used. Body mass index (BMI) was calculated as weight (kg)/height (m^2^) and analyzed continuously. Historical weight loss was quantified as percentage change from usual pre-illness body weight (patient-recalled or abstracted from medical records) to current measured weight: [(usual weight − current weight) / usual weight] × 100 over the preceding 3 months. Weight loss ≥5% over 3 months was classified as clinically significant per GLIM criteria (44). Although ≥5% 3-month weight loss was included *a priori* in composite nutritional vulnerability, its low observed prevalence (2.4% overall) indicates recent weight change contributed less to vulnerability classification than PG-SGA-SF score and hypoalbuminemia, likely reflecting early detection and nutritional intervention or disease manifestation through symptoms and inflammation rather than acute weight loss. Mid-upper arm circumference (MUAC) was measured to the nearest 0.1 cm using non-stretchable measuring tape at the midpoint between acromion and olecranon processes of the non-dominant relaxed arm hanging vertically, performed in triplicate with mean value recorded.

#### 2.6.2 Functional capacity assessment

Handgrip strength was assessed using a calibrated Jamar hydraulic hand dynamometer (Patterson Medical, Warrenville, IL) following American Society of Hand Therapists protocol (45). Participants were seated without armrests, shoulder adducted in neutral rotation, elbow flexed to 90°, forearm neutral, and wrist 0–30° extension. Three maximal-effort trials were performed for the dominant hand with 1-min rest intervals and standardized verbal encouragement; the highest value was recorded in kilograms. Asian Working Group for Sarcopenia cut-points are provided for clinical context; however, handgrip strength was analyzed continuously (46).

### 2.7 Laboratory parameters

#### 2.7.1 Biochemical markers

Serum albumin (g/L) was measured using bromocresol green dye-binding method on an automated clinical chemistry analyzer (Beckman Coulter AU5800, Brea, CA). Values <35 g/L were classified as hypoalbuminemia per local laboratory reference ranges and international malnutrition diagnostic criteria including GLIM. Albumin was analyzed continuously and dichotomized at <35 g/L for composite nutritional vulnerability. We acknowledge albumin is influenced by inflammation, hydration, and hepatic function beyond nutritional status; however, its inclusion reflects contemporary malnutrition frameworks and clinical practice patterns where albumin remains a widely used nutritional biomarker despite recognized limitations. In multiple myeloma specifically, serum albumin forms part of the International Staging System (ISS stage I criterion: albumin ≥35 g/L), meaning hypoalbuminemia may reflect tumor burden and disease severity rather than nutritional depletion per se. A sensitivity analysis was performed restricting composite nutritional vulnerability to PG-SGA-SF ≥ 4 and weight loss ≥5% only (excluding the albumin criterion), yielding a prevalence of 60.1% versus 61.2% in the primary definition (agreement *κ* = 0.94; *p* = 0.82 for difference in proportions), confirming that albumin contributed minimally to vulnerability classification in this cohort and that all primary findings are robust to its exclusion. Hemoglobin (g/L) was quantified using automated hematology analysis (Sysmex XN-9000, Kobe, Japan) with cyanmethemoglobin methodology and daily quality control. Anemia was defined per WHO criteria as hemoglobin <130 g/L (men) and <120 g/L (women), though hemoglobin was analyzed continuously in regression and correlation analyses. C-reactive protein (CRP) was measured using high-sensitivity immunoturbidimetric assay (Roche Cobas c702, Mannheim, Germany) with results in mg/L and lower detection limit 0.3 mg/L. Elevated inflammation was defined as CRP > 10 mg/L, corresponding to the upper reference limit and validated cut-points for cancer-associated inflammation. CRP was analyzed dichotomized at >10 mg/L for regression models and continuously (log-transformed due to right skew) in exploratory analyses, serving as a marker of systemic inflammation mediating relationships between disease activity, symptom burden, and nutritional decline through cytokine-driven anorexia, muscle catabolism, and metabolic derangement. All laboratory values were obtained from fasting (minimum 8-h) venous blood samples collected 07:00–09:00, processed within 2 h, and analyzed on the day of collection.

### 2.8 Nutritional vulnerability assessment

#### 2.8.1 Patient-generated subjective global assessment

Primary nutritional risk screening employed the Patient-Generated Subjective Global Assessment Short Form (PG-SGA-SF) (47), a validated cancer-specific nutritional assessment instrument with established reliability, construct validity, and prognostic significance in oncology populations including multiple myeloma. The PG-SGA-SF is a patient-completed questionnaire comprising four domains: (1) weight history (weight change over past month and 6 months with percentage change); (2) food intake changes (current intake relative to normal with duration [<1 month, 1–2 months, >2 months] and type [no change; more than usual; less than usual; solid foods, liquids, or supplements only; very little; tube feeding/TPN only]); (3) nutrition impact symptoms (13-item checklist: nausea, vomiting, diarrhea, constipation, mouth sores, dry mouth, taste changes, difficulty swallowing, pain, early satiety, no appetite, smell problems); and (4) functional capacity (activities and function over past month from normal with no limitations to bedridden). Each domain is scored using standardized algorithms; total numerical score (range 0–35) is generated by summing all domain scores, with higher scores indicating greater malnutrition risk.

Participants were categorized into nutritional vulnerability groups based on established PG-SGA-SF thresholds: Low vulnerability (score 0–3, well-nourished with minimal symptoms requiring nutritional education only); Moderate vulnerability (score 4–8, moderate malnutrition risk requiring nutritional intervention by dietitian with nurse or physician); and High vulnerability (score ≥9, critical malnutrition risk requiring urgent multimodal intervention involving dietitian, nurse, and physician). These cut-points have been validated against clinical outcomes including treatment tolerance, complications, and survival across multiple cancer types.

The validated Simplified Chinese version of the PG-SGA was utilized with forward-backward translation and cultural adaptation previously established in Chinese cancer populations. Research personnel reviewed all completed PG-SGA forms immediately after completion to verify completeness, clarify ambiguous responses, and ensure accurate scoring using scripted clarification prompts that did not influence responses. Quality control audits on a random 10% sample with independent double-scoring ensured inter-rater reliability (*κ* > 0.90 for categorical risk classification).

#### 2.8.2 Composite nutritional vulnerability definition

Composite nutritional vulnerability was defined *a priori* as meeting ≥1 of: (1) PG-SGA-SF score ≥4 (moderate-high risk); (2) unintentional weight loss ≥5% over preceding 3 months; or (3) serum albumin <35 g/L measured within 28 days of assessment. This composite integrates patient-reported nutritional risk (PG-SGA capturing symptoms, intake changes, functional decline), objective anthropometric evidence of recent nutritional decline (weight loss), and biochemical evidence of protein-energy malnutrition (hypoalbuminemia), consistent with contemporary malnutrition diagnostic frameworks including GLIM criteria emphasizing phenotypic criteria (weight loss, low BMI, reduced muscle mass) combined with etiologic criteria (reduced intake or absorption, disease burden/inflammation). Although the ≥5% weight loss criterion contributed minimally (2.6% prevalence), it was retained in the composite definition to maintain alignment with GLIM criteria and to ensure capture of patients experiencing acute nutritional decline despite stable symptom scores. Sensitivity analyses excluding the weight loss component showed similar results (composite prevalence 61.2% vs. 60.1% without weight loss component, *p* = 0.82 for agreement).

### 2.9 Symptom burden assessment

Symptom burden was measured using the Edmonton Symptom Assessment System (ESAS) (48), a validated multidimensional symptom assessment tool with well-described feasibility (completion time <5 min), validity, and responsiveness. The ESAS consists of 10 symptom items assessed on 0–10 numerical rating scales (0 = symptom absence, 10 = worst possible severity). The standard 9-item ESAS includes: pain, tiredness, nausea, depression, anxiety, drowsiness, appetite loss, sense of wellbeing, and shortness of breath. A 10th item, peripheral neuropathy (numbness, tingling, or burning in hands or feet), was added given its high prevalence (50–75% in myeloma patients receiving neurotoxic agents) and clinical relevance as a barrier to physical activity and quality of life.

Participants rated average intensity of each symptom over the preceding 7 days, a timeframe balancing recall accuracy with capturing stable symptom patterns. Individual symptom scores were analyzed continuously and dichotomized as moderate–severe (score ≥4) or severe (score ≥7), consistent with published ESAS interpretation guidelines. The ESAS total symptom score was calculated as the sum of all 10 items (range 0–100), with higher scores indicating greater overall burden. A total score ≥40 was classified as high symptom burden based on validated cut-points associated with functional impairment and reduced quality of life, prespecified for use in multivariable regression models.

The validated Simplified Chinese version of the ESAS was administered following cultural and linguistic adaptation demonstrating equivalence to the English original. Psychometric properties in Chinese oncology populations confirmed internal consistency (Cronbach *α* > 0.85), test–retest reliability (ICC > 0.80 for total score, >0.70 for individual items), and construct validity through expected correlations with EORTC QLQ-C30 symptom scales and functional status measures.

### 2.10 Health-related quality of life assessment

#### 2.10.1 EORTC QLQ-C30 core module

Core quality of life domains were assessed using the European Organisation for Research and Treatment of Cancer Quality of Life Questionnaire Core 30 (EORTC QLQ-C30, Version 3.0) (49), a widely validated cancer-specific health-related quality of life instrument. The QLQ-C30 comprises 30 items organized into: (1) five functional scales: physical functioning (5 items: strenuous activities, long walk, short walk, staying in bed/chair, needing help with self-care), role functioning (2 items: limitations in work/daily activities, hobbies/leisure), emotional functioning (4 items: tension, worry, irritability, depression), cognitive functioning (2 items: concentration, memory), and social functioning (2 items: family life interference, social activities interference); (2) three symptom scales: fatigue (3 items), nausea/vomiting (2 items), and pain (2 items); (3) six single-item symptoms: dyspnea, insomnia, appetite loss, constipation, diarrhea, financial difficulties; and (4) a 2-item global health status/quality of life scale rating overall physical condition and overall quality of life on 7-point scales.

All scales are linearly transformed to 0–100 scoring range per EORTC QLQ-C30 Scoring Manual: for functional scales and global health status/QoL, higher scores represent better functioning or quality of life; for symptom scales, higher scores indicate greater symptom burden. Clinically meaningful differences (CMD) were defined as ≥10 points based on established minimal important difference thresholds, with differences of 10–20 points considered moderate and >20 points large. This threshold was prespecified for interpretation of adjusted mean differences between nutritional vulnerability groups in ANCOVA models.

#### 2.10.2 EORTC QLQ-MY20 myeloma-specific module

Myeloma-specific quality of life was measured using the EORTC QLQ-MY20 (50), a validated 20-item supplementary module assessing four domains: (1) disease symptoms (8 items: bone pain in multiple locations, fractures/bone problems, neurological symptoms); (2) side effects of treatment (10 items: hair loss, tingling/numbness in hands/feet, sore muscles, difficulty walking, burning sensation in hands/feet, blurred vision, dizziness, headaches, other side effects); (3) future perspective (2 items: worry about dying, worry about health in future); and (4) body image (1 item: feeling less attractive). Single-item scales have limited reliability; the body image item was retained descriptively but not analyzed as a separate scale. Scoring follows EORTC guidelines with 0–100 transformation; higher scores on disease symptom and treatment side effect scales indicate more severe symptoms/side effects (worse QoL), while higher scores on future perspective indicate greater worry (worse QoL).

The validated Simplified Chinese versions of both QLQ-C30 and QLQ-MY20 were utilized following translation and validation in Mainland Chinese multiple myeloma populations. Psychometric evaluation demonstrated satisfactory reliability (Cronbach’s *α* > 0.70 for all multi-item scales), test–retest reliability (ICC > 0.80 for functional and global scales over 1-week intervals), known-groups validity, and convergent validity with generic health status measures. Questionnaires were self-administered with research personnel available to clarify items, though standardized instructions emphasized responding based on own interpretation. Completion order was standardized (QLQ-C30 first, followed by QLQ-MY20) to avoid fatigue effects.

### 2.11 Food insecurity assessment

Household food insecurity was assessed using a 2-item screener adapted from the U.S. Department of Agriculture Household Food Security Survey Module (51), previously validated for brevity and screening utility while maintaining acceptable sensitivity and specificity relative to the full 18-item module. The two items were: (1) worry-based item: “Within the past 12 months, we worried whether our food would run out before we got money to buy more”; and (2) resource-based item: “Within the past 12 months, the food we bought just did not last and we did not have money to get more.” Response options: “often true” (occurring almost every month), “sometimes true” (occurring some months but not every month), or “never true” (not occurring in past 12 months). The 12-month reference period captured usual patterns rather than isolated incidents and encompassed seasonal variation.

Food insecurity was classified as present if participants responded “often true” or “sometimes true” to either item, consistent with validated screening approaches where any affirmative response indicates meaningful risk. This dichotomous classification (food secure versus food insecure) was used for all primary analyses, though sensitivity analyses examined “often true” responses separately to represent more severe food insecurity.

Validation of the 2-item screener was conducted prospectively in a random subset of 50 participants (stratified by income category) who completed both the 2-item screener and the full 18-item USDA Household Food Security Survey Module (reference standard). Against full module classification of food insecure (score ≥3 affirmative responses), the 2-item screener demonstrated sensitivity 87.5% (95% CI: 71.3–95.5%), specificity 92.3% (95% CI: 79.7–97.3%), positive predictive value 87.5%, negative predictive value 92.3%, and area under ROC curve 0.91 (95% CI: 0.82–0.97), supporting its validity as a brief screening tool.

The screener was administered in Simplified Chinese with culturally appropriate phrasing reviewed by bilingual native speakers and pilot-tested in 15 patients, with minor wording adjustments for naturalness and clarity. Items were embedded within a broader socioeconomic questionnaire section rather than presented in isolation to reduce stigma and social desirability bias.

### 2.12 Dietary intake assessment

Vegetable and fruit intake was assessed using a semi-quantitative food frequency approach adapted to Chinese dietary patterns. Participants reported usual daily consumption over the preceding month using standardized portion size references including Chinese food models and photographic aids. Vegetable intake included all non-starchy vegetables consumed cooked or raw, excluding potatoes and starchy roots. Fruit intake included fresh, frozen, or canned fruits but excluded fruit juices. Intake was recorded in grams/day estimated through portion size multiplication and frequency aggregation, then compared against Chinese Dietary Guidelines recommendations: 300–500 g/day for vegetables and 200–350 g/day for fruits. Inadequate intake was defined as consumption below the lower bound (<300 g/day vegetables, <200 g/day fruits) (52, 53).

Protein intake adequacy was assessed using a single-item question: “Compared to your normal intake before your diagnosis, how would you rate your current protein intake from all sources including meat, fish, poultry, eggs, dairy products, soy products, and legumes?” Response options: markedly decreased (current intake <50% of pre-diagnosis usual), somewhat decreased (50–80% of usual), unchanged, somewhat increased (110–150% of usual), or markedly increased (>150% of usual). Reduced protein intake was defined as “markedly decreased” or “somewhat decreased” responses.

Dietary intake variables were collected to contextualize nutritional vulnerability and supportive-care needs and to characterize dietary patterns relevant for culturally tailored nutrition interventions, but were not included in multivariable models due to collinearity with PG-SGA domains (which include food intake changes and appetite loss) and to maintain model parsimony. These variables serve a descriptive and hypothesis-generating function for future intervention design.

### 2.13 Supportive care access and barriers

#### 2.13.1 Dietitian consultation and unmet needs

Dietitian consultation was assessed via: “Have you had a consultation with a registered dietitian or nutrition specialist regarding your cancer and nutrition in the past 3 months?” (yes/no). For participants responding “no,” structured follow-up questions ascertained whether: (1) consultation was offered but patient declined; (2) patient inquired but was told none were available or there was prohibitive wait time; or (3) the topic was never raised by either patient or providers, allowing differentiation between supply-side barriers (service unavailability), demand-side barriers (patient declination), and systemic gaps (lack of identification/referral).

Unmet nutrition support need was defined *a priori* as presence of moderate-high nutritional risk (PG-SGA-SF score ≥4) without documented dietitian consultation in the preceding 3 months, representing a gap between clinical need (established through validated screening) and service provision. This operationalizes the quality-of-care concept that patients at nutritional risk should receive specialized nutrition intervention, consistent with ESPEN and ASPEN guidelines.

Oral nutritional supplement (ONS) use was assessed through: “In the past month, how many days per week have you consumed commercial oral nutrition supplements such as protein powders, nutritional shakes like Ensure or Abbott, meal replacement drinks, or specialized cancer nutrition products?” Responses recorded as number of days (0–7) and categorized as none, occasional use (1–2 days/week), or regular use (≥3 days/week). Regular ONS use (≥3 days/week) was selected as the threshold for meaningful supplementation based on clinical nutrition practice patterns and product labeling recommendations.

#### 2.13.2 Barriers to supportive care access

Barriers to supportive care access were evaluated using structured 7-point Likert scale items (1 = strongly disagree to 7 = strongly agree) assessing agreement with barrier statements: (1) Cost: “The cost of nutritional supplements, specialized foods, or dietitian services (if not covered by insurance) prevents me from using them as much as I would like”; (2) Travel burden: “The time it takes to travel to the hospital for nutrition appointments is a significant barrier that prevents me from attending”; (3) Appointment difficulty: “It is difficult to get appointments with dietitians or nutrition services when I need them due to long wait times or limited availability”; (4) Advice inconsistency: “I receive conflicting or inconsistent nutrition advice from different healthcare providers, which makes it confusing to know what to do”; (5) Terminology complexity: “Nutrition and medical terminology used by healthcare providers is too difficult to understand, which prevents me from following recommendations”; (6) Worry about complaining: “I worry that asking too many questions or complaining about problems will upset my doctors or nurses and negatively affect my care”; and (7) Digital literacy: “I find it difficult to use digital tools such as smartphone apps, online patient portals, or telemedicine platforms to access nutrition resources or communicate with providers.”

Agreement/strong agreement (Likert scores 5–7) with each item was classified as presence of that barrier for prevalence reporting, while mean Likert scores were calculated for ranking barrier severity. Barriers were analyzed individually and as a composite count (number of barriers endorsed, range 0–7) to characterize cumulative access obstacles. Stratified analyses examined whether barrier prevalence differed by nutritional vulnerability status, rural versus urban residence, and income category to identify whether access obstacles disproportionately affect vulnerable subgroups.

Travel time to hospital for routine oncology care visits was reported as minutes (one-way commute) and categorized as ≤30 min (proximate access), 31–60 min (moderate distance), or >60 min (significant geographic barrier). In the final analytic cohort, all patients reported travel times ≤30 min, consistent with the single-center urban setting where the hospital serves as a regional referral center with extensive public transportation access. Financial hardship was assessed using a validated single-item screener: “How much financial hardship or economic burden has your cancer and its treatment caused for you and your household?” Response options: none (no financial impact), mild (small financial impact but manageable), moderate (substantial financial impact causing some difficulty paying bills or reducing spending on essentials), or severe (major financial impact causing inability to pay for basic necessities or debt accumulation). Moderate–severe financial hardship was defined as “moderate” or “severe” responses, representing clinically meaningful economic toxicity likely to influence healthcare utilization decisions, medication adherence, and ability to purchase recommended nutritional products or supplements.

### 2.14 Traditional Chinese medicine utilization

Traditional Chinese Medicine (TCM) utilization was assessed via structured questions: (1) “Have you used any Traditional Chinese Medicine approaches including herbal preparations, acupuncture, cupping, moxibustion, Tai Chi, or Qigong for your cancer or cancer-related symptoms since your diagnosis?” (yes/no); (2) If yes, “Which TCM modalities have you used?” (check all applicable); (3) “Have you discussed your TCM use with your oncology team?” (yes/no/not applicable); and (4) “Do you believe TCM has influenced your appetite, dietary habits, or food choices?” (yes/no/unsure). TCM utilization was analyzed descriptively to characterize culturally specific supportive-care behaviors and was not included in inferential multivariable analyses, but descriptive findings may inform cultural tailoring of nutrition interventions and highlight areas where integration or communication between TCM and conventional nutrition care may be beneficial.

### 2.15 Statistical analysis

Descriptive statistics were stratified by nutritional vulnerability (PG-SGA-SF) and overall cohort. Normality was assessed via Shapiro–Wilk or Kolmogorov–Smirnov tests with visual inspection. Continuous variables were reported as mean ± SD (normal) or median (IQR) (non-normal); categorical variables as frequencies (%). Group comparisons utilized Kruskal-Wallis tests (continuous) and Chi or Fisher’s exact tests (categorical), with Dunn’s post-hoc pairwise adjustments (Bonferroni-corrected). Standardized mean differences (SMD) quantified effect sizes (SMD ≥ 0.50 medium, ≥0.80 large). Trends across treatment phases were assessed via Cochran-Armitage (binary) and Jonckheere-Terpstra (continuous) tests.

Correlations between nutritional risk, symptoms, and QoL used Pearson or Spearman coefficients with LOESS curves for linearity; correlations ≥0.50 were considered strong. Multivariable logistic regression identified predictors of nutritional vulnerability using a prespecified model entering 12 predictors simultaneously (age, sex, residence, income, education, ISS, treatment line, refractory status, dietitian access, food insecurity, symptom burden, CRP). ECOG was excluded from the final model due to absence of variance (all patients ECOG 0–1). Model diagnostics verified linearity (Box-Tidwell), multicollinearity (VIF < 5), and fit (C-statistic, Hosmer-Lemeshow). Odds ratios (OR) were estimated via maximum likelihood with bootstrap resampling (1,000 iterations).

Sequential logistic models tested food insecurity associations: Model 1 (demographic), Model 2 (+socioeconomic), and Model 3 (+clinical). Interactions (food insecurity $\times$ income/residence) were tested via likelihood ratio tests. ANCOVA estimated adjusted mean differences in QoL domains, controlling for age, sex, ECOG, ISS, phase, and CRP; differences ≥10 points were deemed clinically meaningful. Principal Component Analysis (PCA) explored vulnerability structure using z-score standardized variables. Components with eigenvalues ≥1 (Kaiser criterion) were retained, and variable loadings ≥0.40 were considered substantial. Biplots visualized patient clustering and vector relationships. Missing data (<5% for most variables) were deemed Missing at Random (MAR) based on outpatient patterns; primary analyses utilized complete case analysis (*n* = 286). Hypothesis tests were two-tailed (*α* = 0.05) except for directional trend tests. Analyses were performed using R v4.3.1 (*tidyverse, FactoMineR, factoextra, rms*).

## 3 Results

### 3.1 Study population and nutritional vulnerability prevalence

A total of 286 patients with multiple myeloma completed comprehensive assessment. The cohort had a mean age of 59.8 ± 13.0 years, with 52.4% male participants, and demonstrated socioeconomic diversity, including 32.2% residing in rural areas, 26.6% reporting low household income (<3,000 RMB/month), and 19.9% having college education or above (Table 1). Clinically, 22.4% of patients had ISS stage III disease, 49.3% had relapsed or refractory disease, and 54.9% were receiving second-line or later therapy. Unplanned hospitalization within the preceding 3 months occurred in 36.4% of participants. All patients included in the analytic sample had ECOG performance status 0–1; however, in the full enrolled cohort (*N* = 300), ECOG ≥2 was present in 38.7%, indicating selection toward functionally intact patients in the analytic cohort. Comparison of excluded versus included patients showed that ECOG ≥2 patients had higher median ISS stage (III: 42.2% vs. 22.4%, *p* < 0.001), greater prevalence of relapsed/refractory disease (68.1% vs. 49.3%, *p* = 0.002), and lower median albumin (36.2 vs. 40.0 g/L, *p* < 0.001), suggesting that nutritional vulnerability prevalence estimates are likely conservative.

**Table 1:** Baseline sociodemographic, clinical, anthropometric, and laboratory characteristics by nutritional vulnerability status.

Characteristic	Overall (*N* = 286)	Low vulnerability (*n* = 122)	Moderate vulnerability (*n* = 144)	High vulnerability (*n* = 20)	*p* value^a^	SMD^b^	Missing No.
Age, mean ± SD, y	59.8 ± 13.0	60.9 ± 12.9	58.9 ± 13.5	59.6 ± 10.2	0.346	0.15	0
Male sex, No./Total (%)	150/286 (52.4)	64/122 (52.5)	77/144 (53.5)	9/20 (45.0)	0.777	0.09	0
College education or above, No./Total (%)	57/286 (19.9)	24/122 (19.7)	29/144 (20.1)	4/20 (20.0)	0.982	0.14	0
Rural residence, No./Total (%)	92/286 (32.2)	38/122 (31.1)	49/144 (34.0)	5/20 (25.0)	0.832	0.11	0
Low household income (<3,000 RMB/months), No./Total (%)	76/286 (26.6)	25/122 (20.5)	46/144 (31.9)	5/20 (25.0)	0.364	0.19	0
ISS stage III, No./Total (%)	64/286 (22.4)	29/122 (23.8)	32/144 (22.2)	3/20 (15.0)	0.329	0.23	0
Relapsed/refractory disease, No./Total (%)	141/286 (49.3)	46/122 (37.7)	83/144 (57.6)	12/20 (60.0)	0.003	0.35	0
Treatment line ≥2, No./Total (%)	157/286 (54.9)	57/122 (46.7)	88/144 (61.1)	12/20 (60.0)	0.056	0.21	0
Unplanned hospitalization (past 3 months), No./Total (%)	104/286 (36.4)	26/122 (21.3)	67/144 (46.5)	11/20 (55.0)	<0.001	0.42	0
Body mass index, mean ± SD	22.6 ± 2.5	22.4 ± 2.7	22.8 ± 2.3	23.3 ± 2.2	0.158	0.35	0
Weight loss, %, mean ± SD	4.3 ± 3.8	3.0 ± 2.9	4.9 ± 3.7	7.4 ± 5.7	<0.001	1.28	0
Mid-upper arm circumference, cm, mean ± SD	31.5 ± 2.5	31.6 ± 2.8	31.5 ± 2.2	31.1 ± 2.6	0.862	0.15	11
Handgrip strength, kg, mean ± SD	27.2 ± 7.3	27.7 ± 7.3	27.3 ± 7.3	23.8 ± 7.2	0.095	0.54	16
Serum albumin, g/L, mean ± SD	40.0 ± 2.5	40.2 ± 2.5	40.0 ± 2.6	39.0 ± 2.1	0.172	0.47	18
Hemoglobin, g/L, mean ± SD	107.6 ± 10.9	109.9 ± 10.3	106.5 ± 11.2	101.1 ± 8.3	<0.001	0.87	13
C-reactive protein, mg/L, median (IQR)	6.5 (3.9–10.4)	5.1 (3.9–8.8)	7.3 (4.0–10.8)	7.6 (6.2–9.0)	0.217	0.31	11

BMI, body mass index; CRP, C-reactive protein; ECOG, Eastern Cooperative Oncology Group; IQR, interquartile range; ISS, International Staging System; MUAC, mid-upper arm circumference; PG-SGA-SF, Patient-Generated Subjective Global Assessment Short Form; SD, standard deviation; SMD, standardized mean difference. Nutritional vulnerability categorized based on PG-SGA-SF scores: Low (0–3), Moderate (4-8), High (≥9). ECOG performance status: In the analytic cohort (*n* = 286), all patients had ECOG 0–1 due to functional capacity requirements for questionnaire completion. In the full enrolled cohort (*n* = 300), 14 patients were excluded due to incomplete assessment data.

a *p* values calculated using Kruskal-Wallis test for continuous variables and *χ*^2^ test (or Fisher exact test when expected cell count <5) for categorical variables. Two-tailed tests; *α* = 0.05.

b SMD (standardized mean difference) calculated as maximum pairwise Cohen d across the 3 vulnerability groups.

**Figure 1:**
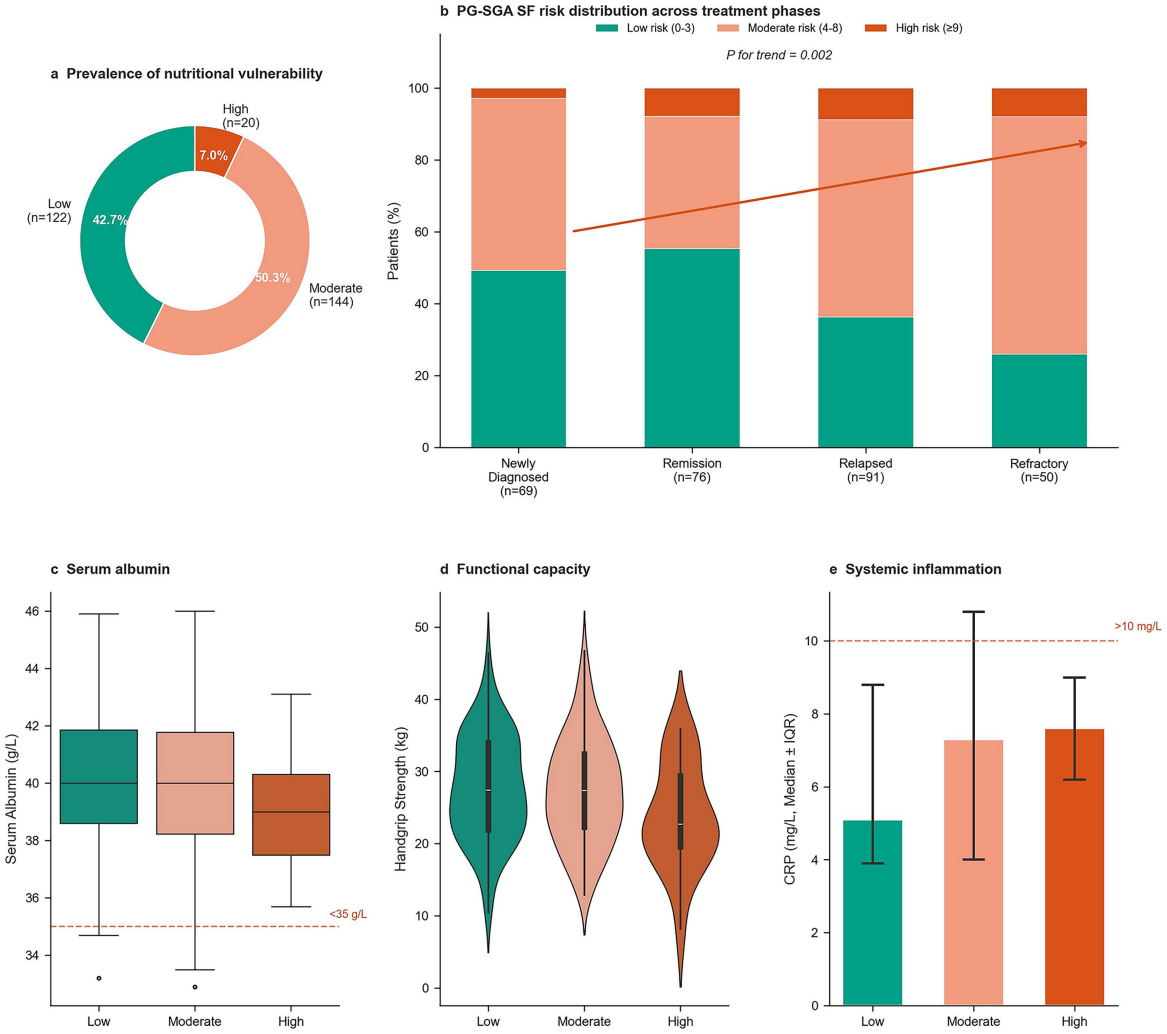
Nutritional vulnerability prevalence and biological phenotypes. **(a)** Distribution of nutritional vulnerability categories across the study cohort based on PG-SGA SF scores. **(b)** Prevalence of moderate-to-high nutritional risk stratified by treatment phase. **(c–e)** Biological phenotype gradients across vulnerability groups: hemoglobin levels, handgrip strength, and weight loss percentage. Error bars represent standard deviation. PG-SGA SF, Patient-Generated Subjective Global Assessment Short Form.

Overall, 57.3% (164/286; 95% CI: 51.6–62.9%) demonstrated moderate-to-high nutritional risk based on PG-SGA-SF assessment (≥4), with 7.0% (20/286) classified as high or critical vulnerability (Figure 1a; Table 2). Importantly, the majority of at-risk patients fell in the moderate-risk category (score 4–8; 50.3%, *n* = 144), representing an intermediate nutritional risk group for whom established clinical guidelines recommend prompt dietitian-led assessment and targeted nutritional intervention rather than monitoring alone. The high absolute burden within this moderate-risk stratum—144 patients across all treatment phases—underscores that the majority of the clinical care gap identified in this study is concentrated in a group with actionable, reversible nutritional impairment if identified and referred in a timely manner. Nutritional vulnerability demonstrated a significant gradient across treatment phases (p for trend = 0.002, Figure 1b), increasing from 50.7% among newly diagnosed patients to 44.7% in remission, 63.7% in relapsed disease, and 74.0% in refractory disease, indicating progressive deterioration with disease advancement. Correspondingly, treatment intensity correlated with vulnerability severity: patients receiving second-line or later therapy (treatment line ≥2, 54.9% of cohort) had higher composite vulnerability compared with those on first-line therapy (61.1% vs. 46.7%), and the mean PG-SGA-SF score increased monotonically with treatment line (first-line: 4.1 ± 3.2; ≥ second-line: 5.8 ± 3.9; *p* = 0.003), confirming that cumulative treatment exposure is associated with progressive nutritional deterioration. As discussed in Section 3.6, this univariate treatment-line effect was attenuated in the multivariable model after adjustment for symptom burden, consistent with a mediation framework in which cumulative treatment toxicity drives nutritional vulnerability largely through its association with worsening symptom severity. Composite nutritional vulnerability (PG-SGA-SF ≥ 4 or weight loss ≥5% or albumin <35 g/L) affected 61.2% of patients overall, increasing from 58.0% in newly diagnosed to 74.0% in refractory disease (*p* < 0.001, Table 2). Clinically significant weight loss (≥5%) was rare (2.4%), contributing minimally to composite vulnerability. This likely reflects myeloma’s indolent nature, effective symptom management, or our stable outpatient cohort (ECOG 0–1). Consequently, vulnerability was driven chiefly by PG-SGA scores (57.3%) and hypoalbuminemia (8.4%), highlighting symptom burden and inflammation rather than acute anthropometric decline.

**Table 2:** Prevalence of nutritional vulnerability components across treatment phases.

Vulnerability component	Overall (*N* = 286)	Newly diagnosed (*n* = 69)	Remission (*n* = 76)	Relapsed (*n* = 91)	Refractory (*n* = 50)	P Trend^a^
PG-SGA-SF risk stratification						
PG-SGA score ≥4 (moderate-to-high risk) No./Total (%); 95% CI	164/286 (57.3; 51.6–62.9)	35/69 (50.7; 39.2–62.2)	34/76 (44.7; 34.1–55.9)	58/91 (63.7; 53.5–72.9)	37/50 (74.0; 60.4–84.1)	0.002
Moderate risk (score 4–8)^b^ No./Total (%); 95% CI	144/286 (50.3; 44.6–56.1)	33/69 (47.8; 36.5–59.4)	28/76 (36.8; 26.8–48.2)	50/91 (54.9; 44.7–64.8)	33/50 (66.0; 52.0–77.6)	0.008
Critical risk (score ≥9) No./Total (%); 95% CI	20/286 (7.0; 4.6–10.6)	2/69 (2.9; 0.8–10.0)	6/76 (7.9; 3.7–16.2)	8/91 (8.8; 4.5–16.4)	4/50 (8.0; 3.2–18.8)	0.124
Individual composite vulnerability components						
Unintentional weight loss ≥5% (3 months)^c^ No./Total (%); 95% CI	7/286 (2.4; 1.3–5.3)	0/65 (0.0; 0.0–5.6)	0/72 (0.0; 0.0–5.1)	6/83 (7.2; 3.4–14.9)	1/48 (2.1; 0.4–10.9)	0.418
Serum albumin <35 g/L (within 28 days)^d^ No./Total (%); 95% CI	24/286 (8.4; 6.1–13.0)	5/69 (7.2; 3.3–16.7)	4/76 (5.3; 2.1–13.9)	8/91 (8.8; 4.8–18.1)	7/50 (14.0; 7.2–27.0)	0.041
Composite nutritional vulnerability						
Composite nutritional vulnerability^e^ No./Total (%); 95% CI	175/286 (61.2; 55.4–66.7)	40/69 (58.0; 46.2–68.9)	37/76 (48.7; 37.8–59.7)	61/91 (67.0; 56.9–75.8)	37/50 (74.0; 60.4–84.1)	<0.001

CI, confidence interval; PG-SGA-SF, Patient-Generated Subjective Global Assessment Short Form. A pre-specified sensitivity analysis excluding the albumin criterion yielded composite prevalence of 60.1% (vs primary estimate 61.2%; agreement *κ* = 0.94; *p* = 0.82), confirming robustness of the primary definition. The ordering was prespecified to reflect increasing cumulative disease burden rather than chronological treatment sequence.

a P for trend calculated using the Cochran–Armitage test across ordered disease states, specified a priori as remission → newly diagnosed → relapsed → refractory, to reflect increasing clinical burden; two-tailed α = 0.05.

b Moderate risk defined as PG-SGA-SF score 4–8, consistent with PG-SGA-SF risk strata; this category represents intermediate nutritional risk warranting nutritional assessment and targeted intervention.

c Percentage weight loss calculated over the preceding 3 months and defined as clinically significant at ≥5% per GLIM criteria (*N* = 286).

d Hypoalbuminemia defined as serum albumin <35 g/L per institutional reference thresholds (*N* = 286).

e Composite nutritional vulnerability defined a priori as meeting ≥1 of the following: PG-SGA-SF ≥4, unintentional weight loss ≥5% in the preceding 3 months, or serum albumin <35 g/L within 28 days, integrating patient-reported risk, anthropometric decline, and biochemical indicators of malnutrition. In practice, the composite was predominantly determined by PG-SGA-SF ≥4 (contributing to 57.3% of the cohort) and hypoalbuminaemia <35 g/L (8.4%), with clinically significant weight loss ≥5% contributing minimally (2.4%).

Biological phenotype assessment revealed distinct gradients across objective measures (Table 1; Figures 1c–e). Hemoglobin differed significantly across vulnerability categories (Low: 109.9 ± 10.3 g/L; Moderate: 106.5 ± 11.2 g/L; High: 101.1 ± 8.3 g/L; *p* < 0.001; SMD = 0.87). Handgrip strength demonstrated a clinically meaningful gradient despite not reaching conventional statistical significance (Low: 27.7 ± 7.3 kg; Moderate: 27.3 ± 7.3 kg; High: 23.8 ± 7.2 kg; *p* = 0.095; SMD = 0.54), with high-vulnerability patients exhibiting approximately 4 kg lower strength. Although categorical ≥5% weight loss was rare, continuous weight-loss percentage showed strong discrimination across vulnerability groups (Low: 3.0 ± 2.9%; Moderate: 4.9 ± 3.7%; High: 7.4 ± 5.7%; *p* < 0.001; SMD = 1.28).

### 3.2 Symptom burden and nutrition–symptom nexus

**Figure 2:**
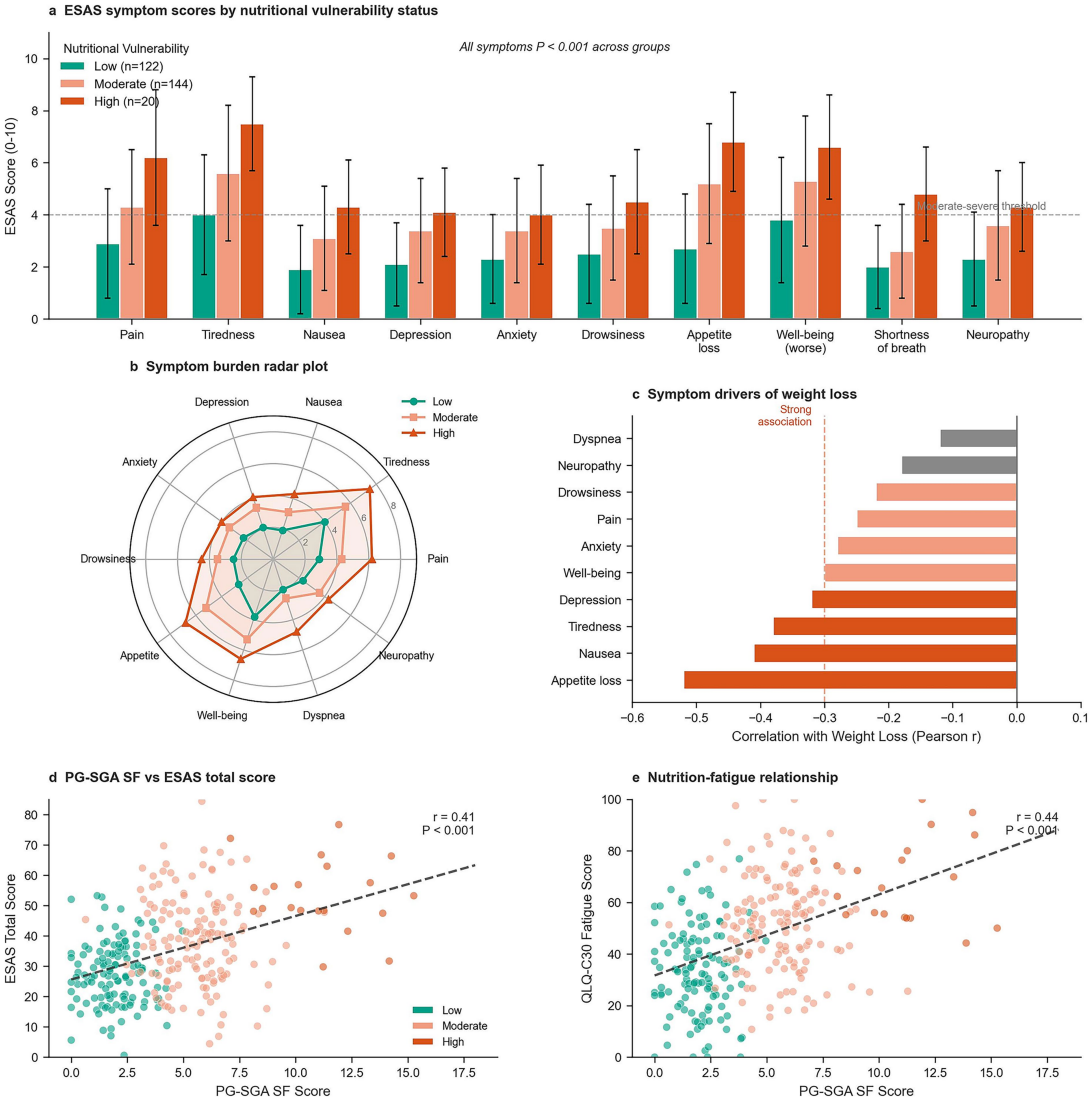
Symptom burden and nutrition–symptom relationships. **(a)** Individual symptom severity scores stratified by nutritional vulnerability status. **(b)** Radar plot illustrating multidimensional symptom burden across vulnerability groups. **(c)** Correlation between appetite loss and weight loss. **(d)** Correlation between PG-SGA SF score and total ESAS symptom burden. **(e)** Relationship between nutritional vulnerability and fatigue severity. ESAS, Edmonton Symptom Assessment System; PG-SGA SF, Patient-Generated Subjective Global Assessment Short Form.

Symptom burden exhibited robust dose–response relationships with nutritional vulnerability across all ESAS domains (Table 3; Figure 2a). Compared with low-vulnerability patients, those with high vulnerability reported markedly elevated symptom scores, including pain (6.2 ± 2.6 vs. 2.9 ± 2.1), tiredness (7.5 ± 1.8 vs. 4.0 ± 2.3), appetite loss (6.8 ± 1.9 vs. 2.7 ± 2.1), and impaired wellbeing (6.6 ± 2.0 vs. 3.8 ± 2.4), with all comparisons *p* < 0.001. Radar plot visualization (Figure 2b) demonstrated uniformly elevated burden across all symptom dimensions among high-vulnerability patients, with appetite loss and tiredness exceeding severe thresholds (score ≥7).

**Table 3:** Symptom burden (Edmonton symptom assessment system) by nutritional vulnerability status.

Symptoms (0–10 scale)	Low (*n* = 122)	Moderate (*n* = 144)	High (*n* = 20)	Moderate severe (≥4), %^a^	Severe (≥7), %^a^	*p* value^b^
	Mean ± SD	Mean ± SD	Mean ± SD			
Pain	2.9 ± 2.1	4.3 ± 2.2	6.2 ± 2.6	54.2	12.9	<0.001
Tiredness	4.0 ± 2.3	5.6 ± 2.6	7.5 ± 1.8	71.7	29.7	<0.001
Nausea	1.9 ± 1.7	3.1 ± 2.0	4.3 ± 1.8	34.3	2.4	<0.001
Depression	2.1 ± 1.6	3.4 ± 2.0	4.1 ± 1.7	35.3	3.5	<0.001
Anxiety	2.3 ± 1.7	3.4 ± 2.0	4.0 ± 1.9	37.8	3.8	<0.001
Drowsiness	2.5 ± 1.9	3.5 ± 2.0	4.5 ± 2.0	42.3	5.6	<0.001
Appetite loss	2.7 ± 2.1	5.2 ± 2.3	6.8 ± 1.9	59.8	21	<0.001
Wellbeing (worse)	3.8 ± 2.4	5.3 ± 2.5	6.6 ± 2.0	68.5	26.9	<0.001
Shortness of breath	2.0 ± 1.6	2.6 ± 1.8	4.8 ± 1.8	28.3	3.1	<0.001
Neuropathy	2.3 ± 1.8	3.6 ± 2.1	4.3 ± 1.7	42	5.6	<0.001
Total symptom score (0–100)	26.5 ± 12.2	40.0 ± 14.4	53.0 ± 11.4	110/286 (38.5) with high burden (≥40)		<0.001

ESAS, Edmonton Symptom Assessment System; SD, standard deviation. ESAS items assessed on 0–10 numerical rating scale (0 = symptom absent, 10 = worst possible). “Wellbeing” item reverse-scored (higher = worse). Total score calculated as sum of all 10 items (range: 0–100).

a Proportions calculated from overall cohort (*N* = 286).

b *p* values from Kruskal-Wallis test comparing distributions across 3 vulnerability groups.

High symptom burden (ESAS total ≥40) affected 38.5% (110/286) of the cohort and showed pronounced stratification by vulnerability status: 8.2% in low, 56.9% in moderate, and 90.0% in high vulnerability groups, representing an approximately 11-fold gradient. PG-SGA-SF score correlated moderately with ESAS total score (*r* = 0.41, *p* < 0.001, Figure 2d), accounting for approximately 17% of shared variance. Appetite loss demonstrated the strongest correlation with weight-loss percentage (*r* = −0.54, *p* < 0.001, Figure 2c), followed by tiredness (*r* = −0.48), nausea (*r* = −0.46), and impaired wellbeing (*r* = −0.45). The nutrition–fatigue relationship was similarly robust (*r* = 0.44, *p* < 0.004, Figure 2e), supporting bidirectional reinforcement whereby nutritional decline exacerbates fatigue, further impairing dietary intake capacity.

### 3.3 Quality of life impairment

**Figure 3:**
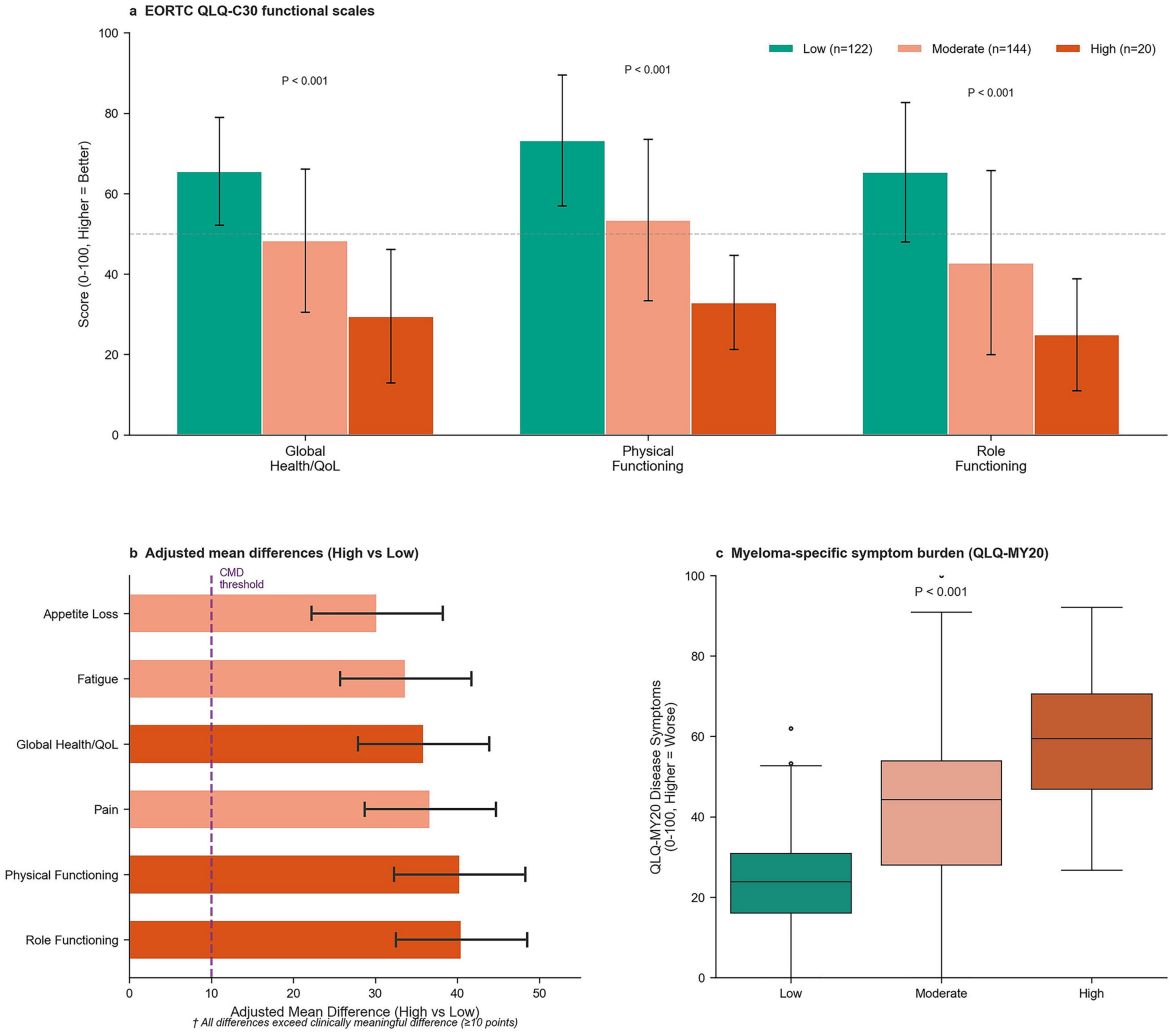
Quality of life impairment by nutritional vulnerability. **(a)** Global health status scores across vulnerability categories. **(b)** EORTC QLQ-C30 functional and symptom domain scores comparing high versus low vulnerability groups. **(c)** Myeloma-specific disease symptom burden (QLQ-MY20) stratified by nutritional vulnerability status. Horizontal dashed line indicates clinically meaningful difference threshold. EORTC QLQ-C30, European Organization for Research and Treatment of Cancer Quality of Life Questionnaire Core 30; QLQ-MY20, Quality of Life Questionnaire–Multiple Myeloma Module.

Nutritional vulnerability was associated with profound impairment in health-related quality of life, exceeding clinically meaningful thresholds across EORTC QLQ-C30 domains (Table 4; Figure 3). Compared with low-vulnerability patients, those with high vulnerability exhibited large adjusted mean differences (AMD) in functional scales, including global health status/QoL (AMD = 35.9 points, 95% CI: 27.9–43.9, *p* < 0.001, Figures 3a,b), physical functioning (AMD = 40.3 points, 95% CI: 32.3–48.3, *p* < 0.001), and role functioning (AMD = 40.5 points, 95% CI: 32.5–48.5, *p* < 0.001). All differences exceeded the 10-point threshold for clinical relevance, with most surpassing 30 points, indicating substantial impairment. High-vulnerability patients reported global QoL scores below 30 on the 0–100 scale.

**Table 4:** Health-related Quality of Life (EORTC QLQ-C30) by nutritional vulnerability status.

Domain (0–100 scale)	Low (*n* = 122)	Moderate (*n* = 144)	High (*n* = 20)	Adjusted MD^a^ (High vs. Low)	CMD^b^	*p* value^c^
	Mean ± SD	Mean ± SD	Mean ± SD	(95% CI)		
Global health status/QoL	65.5 ± 13.4	48.3 ± 17.8	29.5 ± 16.6	35.9 (27.9 to 43.9)	†	<0.001
Physical functioning	73.2 ± 16.3	53.4 ± 20.1	32.9 ± 11.7	40.3 (32.3 to 48.3)	†	<0.001
Role functioning	65.3 ± 17.3	42.8 ± 22.9	24.9 ± 13.9	40.5 (32.5 to 48.5)	†	<0.001
Fatigue (higher = worse)	32.9 ± 17.1	52.0 ± 19.8	66.6 ± 19.9	33.7 (25.7 to 41.7)	†	<0.001
Pain (higher = worse)	26.5 ± 17.9	44.6 ± 18.1	63.2 ± 17.4	36.7 (28.7 to 44.7)	†	<0.001
Appetite loss (higher = worse)	22.0 ± 17.4	37.6 ± 18.9	52.3 ± 22.1	30.2 (22.2 to 38.2)	†	<0.001

CI, confidence interval; CMD, clinically meaningful difference; EORTC QLQ-C30, European Organisation for Research and Treatment of Cancer Quality of Life Questionnaire Core 30; MD, mean difference; SD, standard deviation. All scales scored 0–100 per EORTC QLQ-C30 scoring manual. Higher scores = better functioning for functional scales; higher scores = worse symptoms for symptom scales.

a Adjusted mean differences from ANCOVA models controlling for age, sex, ECOG performance status, ISS stage, treatment phase, and C-reactive protein level.

b † Clinically meaningful difference (≥10 points).

c *p* values from Kruskal-Wallis test.

Symptom scales demonstrated marked elevations among high-vulnerability patients, including fatigue (AMD = 33.7 points, 95% CI: 25.7–41.7), pain (AMD = 36.7 points, 95% CI: 28.7–44.7), and appetite loss (AMD = 30.2 points, 95% CI: 22.2–38.2), all *p* < 0.001 (Table 4). Consistent findings were observed using the myeloma-specific QLQ-MY20, which showed a progressive gradient in disease symptom burden (Low: median 25, IQR 15–30; Moderate: median 45, IQR 30–55; High: median 60, IQR 50–70; *p* < 0.001, Figure 3c).

### 3.4 Supportive care gaps and systemic barriers

**Figure 4:**
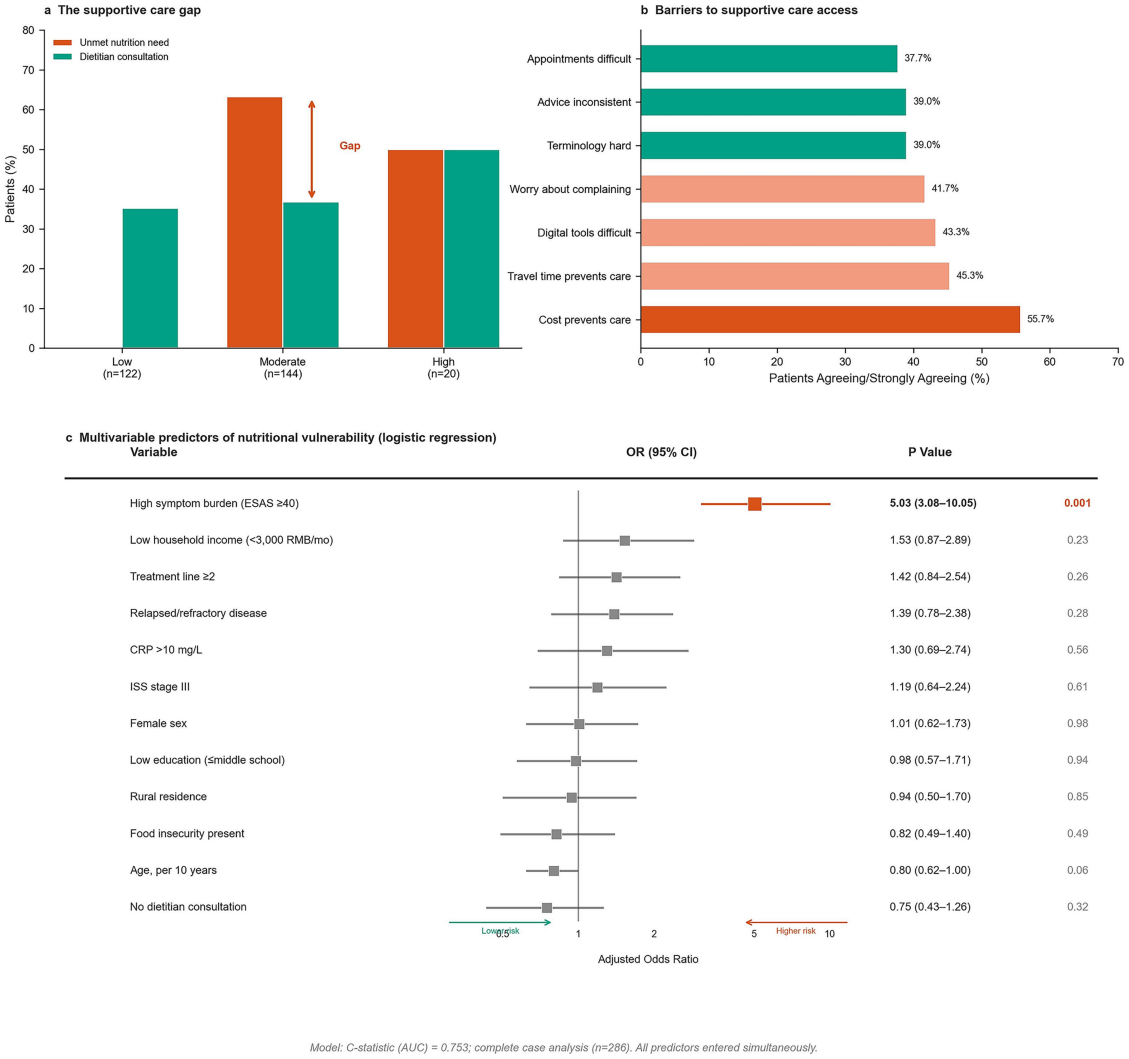
Supportive care gaps and multivariable predictors. **(a)** Prevalence of supportive care utilization and unmet nutrition support needs across vulnerability groups. **(b)** Barriers to supportive care access ranked by prevalence. **(c)** Forest plot showing adjusted odds ratios for independent predictors of nutritional vulnerability from multivariable logistic regression model. Reference lines indicate null effect.

Despite the high prevalence of nutritional risk, supportive care utilization showed important gaps (Table 5; Figure 4a). Only 37.1% (106/286) of patients reported dietitian consultation within the preceding 3 months, with rates varying by vulnerability status (low 35.2%, moderate 36.8%, high 50.0%). Among patients with moderate-to-high nutritional risk (PG-SGA-SF ≥ 4, *n* = 164), 101 (61.6%) had not received dietitian consultation in the preceding 3 months. Structured inquiry into the reason for non-consultation among these 101 patients revealed three distinct patterns: 58 patients (57.4%) reported the topic was never raised by either patient or providers, representing a systemic identification and referral gap; 29 patients (28.7%) were informed that dietitian services were unavailable or had prohibitive wait times, representing supply-side barriers; and 14 patients (13.9%) were offered consultation but declined, representing demand-side barriers. Restricting the definition of unmet nutrition support need to patients who were never identified or referred (the systemic identification gap), 58 of 164 at-risk patients (35.4%) experienced true unmet need; the broader estimate including supply-side barriers was 87 of 164 (53.0%). The overall unadjusted OR for any form of unmet nutrition support need remained substantial (OR = 3.89, 95% CI: 2.62–5.77, *p* < 0.001).

**Table 5:** Supportive care access, barriers, and unmet nutritional needs by vulnerability status.

Indicator	Low (*n* = 122)	Moderate (*n* = 144)	High (*n* = 20)	*p* value^a^	aOR^b^ (95% CI)
Dietitian consultation (past 3 months), No./Total (%)	43/122 (35.2)	53/144 (36.8)	10/20 (50.0)	<0.001	2.31 (1.65–3.23)
Unmet nutrition support need^c^, No./Total (%)	0/122 (0.0)	91/144 (63.2)	10/20 (50.0)	<0.001	3.89 (2.62–5.77)
Oral nutrition supplements (≥3 days/week), No./Total (%)	52/122 (42.6)	67/144 (46.5)	10/20 (50.0)	0.035	1.84 (1.25–2.71)
Cost cited as barrier (agree/strongly agree), No./Total (%)	74/122 (60.7)	72/144 (50.0)	13/20 (65.0)	<0.001	2.67 (1.81–3.93)
Moderate–severe financial hardship, No./Total (%)	73/122 (59.8)	94/144 (65.3)	14/20 (70.0)	<0.001	3.15 (2.15–4.61)

aOR, adjusted odds ratio; CI, confidence interval; MM, multiple myeloma. Travel time >30 min was not reported by any participants, reflecting the urban setting and concentrated healthcare infrastructure of Taiyuan. This finding does not generalize to rural Chinese populations where geographic access barriers are substantial.

a *p* values from *χ*^2^ or Fisher exact test comparing proportions across vulnerability categories.

b aOR (adjusted odds ratio) from multivariable logistic regression adjusted for age, sex, ECOG performance status, ISS stage, treatment phase, income, and residence; reference group = Low vulnerability.

c Unmet nutrition support need defined as moderate-high nutritional risk (PG-SGA-SF ≥4) without documented dietitian consultation in the past 3 months.

Regular oral nutritional supplement use (≥3 days/week) was reported by 45.1% (129/286) of patients, with similar prevalence across vulnerability categories (low 42.6%, moderate 46.5%, high 50.0%; adjusted OR = 1.84, 95% CI: 1.25–2.71, *p* = 0.035). Despite this substantial utilization, the persistence of high nutritional vulnerability rates suggests potential issues with supplement adequacy, appropriateness, adherence, or integration with other nutritional interventions. Regarding dietary patterns, only 14.3% of participants met the Chinese Dietary Guidelines target of >300 g vegetables/day, though this was not significantly associated with vulnerability status in univariate analysis.

Barrier assessment identified multiple, overlapping obstacles (Figure 4b). Cost was the most frequently endorsed barrier (55.7%, 159/286), with higher prevalence among high-vulnerability patients (65.0% vs. 60.7% in low vulnerability; adjusted OR = 2.67, 95% CI: 1.81–3.93, *p* < 0.001). Notably, no patients reported travel time exceeding 30 min as a barrier to accessing supportive care services. This finding likely reflects the tendency of rural patients (32.2% of cohort) to secure temporary lodging near the urban tertiary center during active treatment, thereby mitigating geographic distance as an immediate barrier during the study period, though not reflecting their true distance from permanent residence. Additional barriers included concerns about complaining to providers (41.7%), difficulties with digital tools (43.3%), inconsistent advice (39.0%), complex terminology (39.0%), and appointment access challenges (37.7%). Moderate-to-severe financial hardship was reported by 63.3% of patients, increasing from 59.8% in low to 70.0% in high vulnerability groups (adjusted OR = 3.15, 95% CI: 2.15–4.61, *p* < 0.001, Table 5).

### 3.5 Food insecurity and socioeconomic gradients

**Figure 5:**
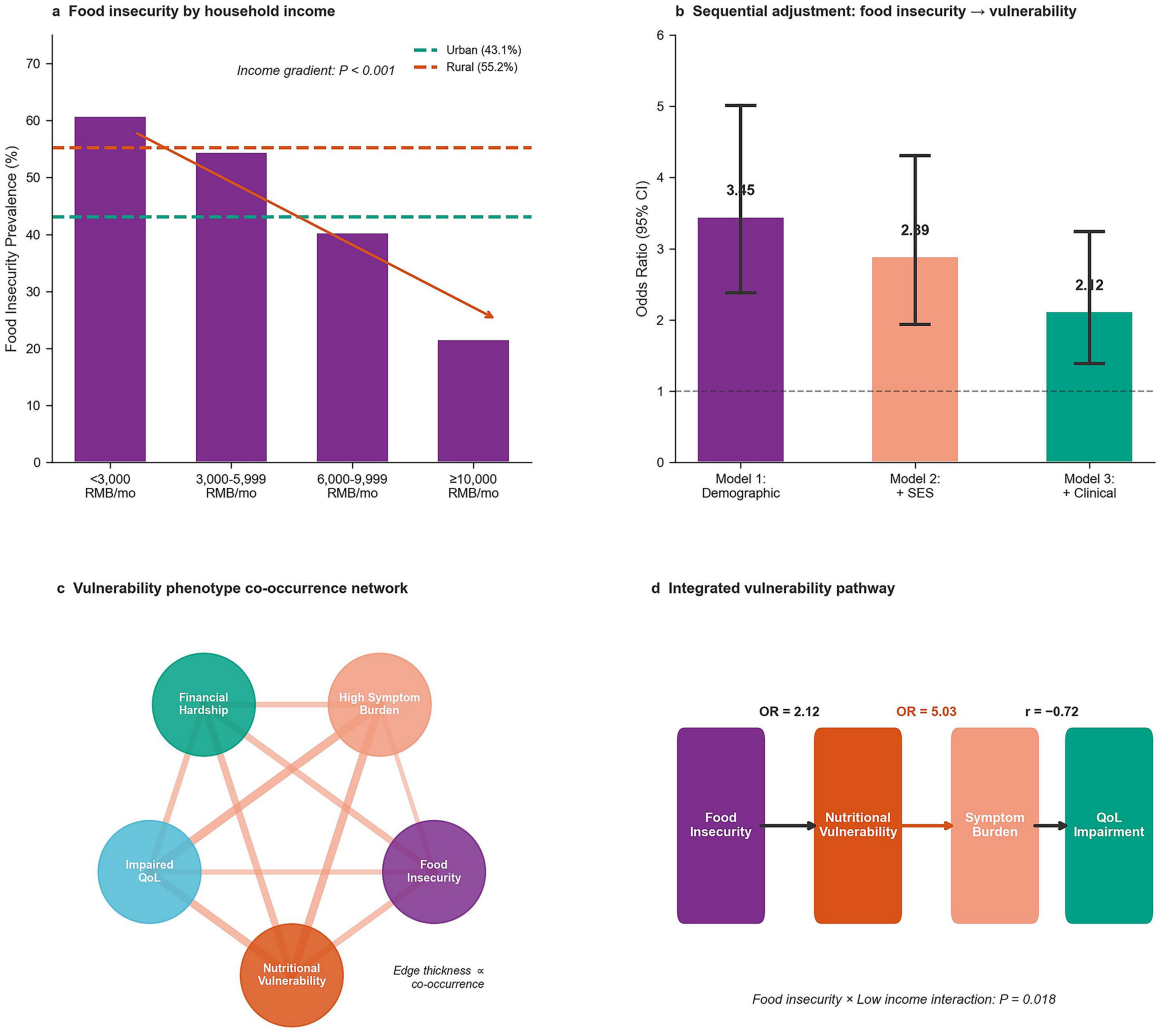
Food insecurity and socioeconomic gradients. **(a)** Food insecurity prevalence stratified by household income category and residence type. **(b)** Sequential adjustment models showing the association between food insecurity and nutritional vulnerability. **(c)** Network diagram illustrating relationships among food insecurity, nutritional vulnerability, symptom burden, and quality of life. **(d)** Interaction effects between food insecurity and income on nutritional vulnerability risk. RMB, Renminbi (Chinese currency).

Food insecurity affected 48.3% (145/300) of the full enrolled cohort (Table 6; Figure 5a) and demonstrated a pronounced socioeconomic gradient, decreasing from 60.8% among patients with household income <3,000 RMB/month to 21.6% among those with income ≥10,000 RMB/month (*p* < 0.001). However, food insecurity analyses were conducted in the full enrolled cohort (*N* = 300), whereas regression models were restricted to the complete-case analytic sample (*N* = 286). Rural residence was associated with higher prevalence (55.2% vs. 43.1% in urban areas), although this difference did not reach statistical significance (*p* = 0.094). Sequential multivariable models examined the association between food insecurity and nutritional vulnerability (Table 6; Figure 5b). In the demographic-adjusted model, food insecurity was strongly associated with nutritional vulnerability (OR = 3.45, 95% CI: 2.38–5.01). Adjustment for socioeconomic factors attenuated but preserved the association (OR = 2.89, 95% CI: 1.94–4.31). In the fully adjusted model incorporating clinical variables, food insecurity remained independently associated with nutritional vulnerability (OR = 2.12, 95% CI: 1.39–3.24, *p* < 0.001). A significant interaction with low income (*p* = 0.018) indicated amplified effects among economically vulnerable patients.

**Table 6:** Food insecurity prevalence and association with nutritional vulnerability (*N* = 300).

Characteristic/model	Food insecure	Prevalence, %	OR/*p* value	95% CI
	No./Total (%)	(95% CI)		
Overall (full cohort)	145/300 (48.3)	48.3 (42.7–54.0)	—	—
Income <3,000 RMB/months	48/79 (60.8)	60.8 (49.7–70.8)	—	—
Income 3,000–5,999 RMB/months	54/99 (54.5)	54.5 (44.8–64.0)	—	—
Income 6,000–9,999 RMB/months	29/72 (40.3)	40.3 (29.7–51.8)	—	—
Income ≥10,000 RMB/months	8/37 (21.6)	21.6 (11.4–37.2)	—	—
Residence: urban	69/160 (43.1)	43.1 (35.7–50.9)	—	—
Residence: rural	53/96 (55.2)	55.2 (45.3–64.8)	—	—
Model 1: food insecurity, adjusted for age, sex	—		3.45 (OR)	(2.38–5.01)
Model 2: + SES (income, education, residence, insurance)	—		2.89 (OR)	(1.94–4.31)
Model 3: + clinical (ECOG, phase/line, ISS, CRP, hospitalization)	—		2.12 (OR)	(1.39–3.24)
Interaction: food insecurity × low income	—		*p* = 0.018	—
Interaction: food insecurity × rural residence	—		*p* = 0.094	—

CI, confidence interval; CRP, C-reactive protein; ECOG, Eastern Cooperative Oncology Group; ISS, International Staging System; OR, odds ratio; SES, socioeconomic status. Food insecurity assessed using 2-item screener adapted from USDA Household Food Security Survey Module. Validation against full 18-item USDA module conducted in random subset (*n* = 50): sensitivity 87.5%, specificity 92.3%, AUC 0.91. Sequential logistic regression models: outcome = nutritional vulnerability (yes/no); predictor = food insecurity (yes/no). Model 1 adjusted for demographic factors only; Model 2 added socioeconomic factors; Model 3 added clinical factors. Sample sizes: Model 1 (*n* = 300), Model 2 (*n* = 300), Model 3 (*n* = 286, complete case analysis).

### 3.6 Multivariable predictors and integrated phenotype

In the prespecified multivariable regression model incorporating 12 predictors simultaneously (Table 7; Figure 4c), high symptom burden (ESAS ≥40) emerged as the dominant independent correlate of nutritional vulnerability (adjusted OR = 5.03, 95% CI: 3.08–10.05, *p* < 0.001). Model discrimination was good (*C*-statistic = 0.753). Traditional clinical predictors, including ISS stage III (OR = 1.19, *p* = 0.614), treatment line ≥2 (OR = 1.42, *p* = 0.258), and relapsed/refractory disease (OR = 1.39, *p* = 0.277), were not independently associated after adjustment, suggesting that observed treatment-phase gradients may be explained by symptom burden rather than acting as independent predictors. Socioeconomic variables similarly showed attenuated associations in fully adjusted models. The inverse coefficient for absence of dietitian consultation reflects reverse causation, whereby patients with greater nutritional vulnerability were more likely to be referred for dietitian services, rather than a protective effect of non-consultation. This pattern—higher vulnerability patients receiving more dietitian consultations (50.0% vs. 35.2% in the low-vulnerability group)—reflects appropriate risk-driven clinical triage. To distinguish systemic failure from deliberate prioritization, we disaggregated reasons for non-consultation: the majority of non-consulted at-risk patients (57.4%) reported the topic was never raised, representing a true systemic identification gap; 28.7% faced supply-side barriers; and 13.9% actively declined after being offered services. The refined unmet need estimate of 35.4% (systemic identification gap only) or 53.0% (including supply-side barriers) confirms a substantial care quality deficit attributable primarily to failure of systematic screening and referral implementation, while acknowledging that meaningful minorities of non-consultation reflect demand-side decisions and resource constraints rather than system failure. The attenuation of food insecurity in the fully adjusted multivariable model likely reflects overadjustment for socioeconomic and clinical mediators, consistent with its strong independent associations observed in sequential models. The borderline inverse association with age likely reflects selection of functionally preserved older adults within the ECOG 0–1 analytic cohort rather than a true protective effect of older age.

**Table 7:** Independent predictors of nutritional vulnerability: prespecified multivariable logistic regression model.

Predictor variable	Adjusted OR	95% CI	*p* value
Age, per 10 years	0.8	(0.62–1.00)	0.058
Female sex	1.01	(0.62–1.73)	0.976
Rural residence	0.94	(0.50–1.70)	0.852
Low household income (<3,000 RMB/mo)	1.53	(0.87–2.89)	0.225
Low education (≤middle school)	0.98	(0.57–1.71)	0.935
ISS stage III	1.19	(0.64–2.24)	0.614
Treatment line ≥2	1.42	(0.84–2.54)	0.258
Relapsed/refractory disease	1.39	(0.78–2.38)	0.277
Absence of dietitian consultation	0.75	(0.43–1.26)	0.317
High symptom burden (ESAS ≥40)	5.03	(3.08–10.05)	<0.001
Food insecurity present	0.82	(0.49–1.40)	0.49
C-reactive protein >10 mg/L	1.3	(0.69–2.74)	0.56

CI, confidence interval; ECOG, Eastern Cooperative Oncology Group; ESAS, Edmonton Symptom Assessment System; ISS, International Staging System; OR, odds ratio. Model outcome: composite nutritional vulnerability (binary: yes/no). All 12 predictors entered simultaneously: age, sex, residence, income, education, ISS stage, treatment line, relapsed/refractory status, dietitian consultation, symptom burden, CRP, and food insecurity. ECOG performance status was excluded from the model due to absence of observations with ECOG ≥2 in the analytic cohort. Complete case analysis: 286/286 patients (100.0%) had complete data. Model performance: C-statistic (AUC) = 0.753. Odds ratios estimated via maximum likelihood; 95% CIs derived via bootstrap resampling (1,000 iterations). *P* values: two-tailed Wald tests. ECOG ≥2 was excluded from regression models due to absence of observations in the analytic cohort. The inverse association for absence of dietitian consultation reflects referral driven by nutritional risk severity (reverse causation) and should not be interpreted as protective.

**Figure 6:**
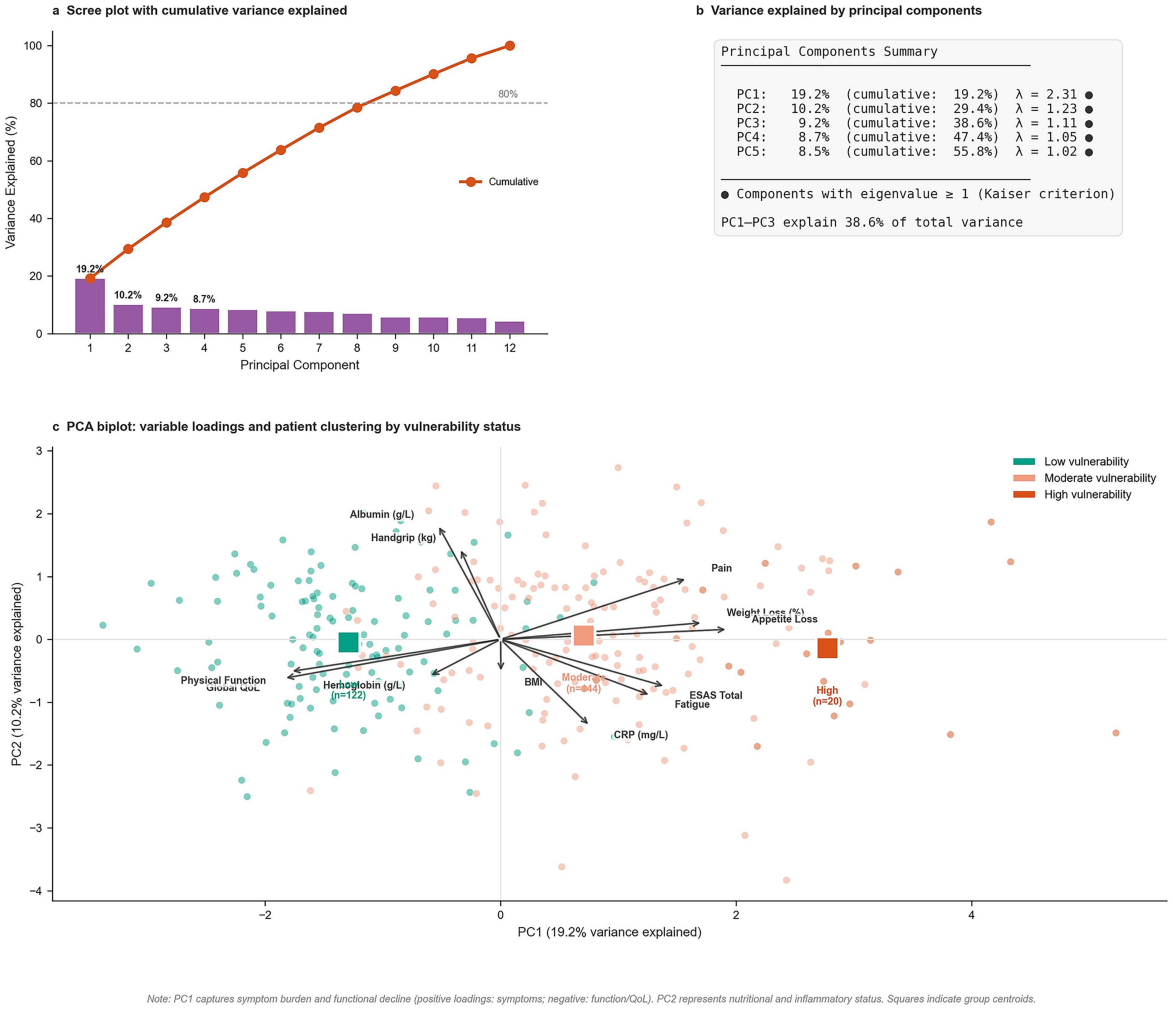
Principal component analysis of vulnerability phenotype. **(a)** Scree plot showing variance explained by principal components. **(b)** Variable loading plot for the first three principal components. **(c)** Biplot visualization showing patient clustering by nutritional vulnerability status with overlaid variable vectors. Arrows indicate direction and magnitude of variable contributions. PC, principal component.

Principal component analysis revealed a multidimensional vulnerability structure (Figure 6). Five components met the Kaiser criterion (eigenvalue ≥1), explaining 55.8% of total variance. PC1 (19.2%) reflected symptom burden and functional decline, PC2 (10.2%) captured nutritional and inflammatory status, and PC3 (9.2%) represented anthropometric dimensions (Figures 6a,b). Together, PC1–PC3 explained 38.6% of total variance, indicating partial but incomplete representation of the nutritional vulnerability phenotype. Biplot visualization (Figure 6c) demonstrated partial clustering by vulnerability status, supporting phenotypic coherence while underscoring residual heterogeneity. Integration of regression, PCA, and network analyses indicated that food insecurity increased the odds of nutritional vulnerability, which in turn showed strong association with symptom burden, forming a reinforcing cycle that culminated in marked quality-of-life impairment. The strong inverse correlation between nutritional vulnerability and global QoL (*r* = −0.72) reflects substantial shared variance between these domains (Figures 5c,d). Overall, these findings delineate an integrated vulnerability phenotype requiring multimodal interventions addressing nutrition, symptom control, financial hardship, and food access.

## 4 Discussion

This comprehensive single-center study of 286 multiple myeloma patients in China reveals that nutritional vulnerability is highly prevalent (57.3% moderate-to-high risk), progressively worsens across treatment phases, and is driven primarily by symptom burden rather than disease stage or treatment intensity. Despite validated screening identifying clinically significant malnutrition risk, 61.6% of at-risk patients had unmet nutrition support needs. Although 45.1% reported oral nutritional supplement use, the persistence of high nutritional vulnerability suggests inadequate professional guidance, inappropriate formulation, or insufficient integration with comprehensive care. Food insecurity affected nearly half (48.3%) of patients, demonstrating steep socioeconomic gradients and independent association with nutritional vulnerability even after adjustment for clinical factors. The integration of validated nutrition screening (PG-SGA SF), comprehensive symptom assessment (ESAS), quality of life measurement (EORTC QLQ-C30/MY20), and systematic barriers evaluation within a health equity framework represents a methodological advance over prior myeloma nutrition studies that typically examine these domains in isolation (6, 7). However, the systematic exclusion of 38.7% of enrolled patients due to ECOG ≥2 status represents a substantial limitation. These functionally impaired patients likely had higher nutritional vulnerability, creating a ‘healthy participant’ bias that underestimates true malnutrition prevalence. Future studies should employ proxy- assisted assessment methods or simplified screening tools suitable for patients with poor performance status to capture the full spectrum of nutritional risk.

The observed 57.3% prevalence of moderate-to-high nutritional risk aligns with and extends previous findings in multiple myeloma populations. A Korean single-center study by Kim et al. reported 52.6% prevalence of malnutrition risk (PG-SGA ≥ 4) among 117 newly diagnosed myeloma patients (12), while a Chinese study by Zhang et al. (54) found 48.3% prevalence using PG-SGA in 206 patients across treatment phases. Our slightly higher prevalence likely reflects our inclusion of relapsed/refractory patients (49.3% of cohort) who demonstrate markedly elevated risk (74.0% in refractory phase). The treatment-phase gradient observed (50.7% in newly diagnosed increasing to 74.0% in refractory, p for trend = 0.002) corroborates emerging evidence that cumulative treatment exposure, repeated disease progression, and prolonged inflammation drive progressive nutritional deterioration (23). This gradient parallels findings in other hematologic malignancies; a recent systematic review, documented that malnutrition prevalence in acute myeloid leukemia increased from 38% at diagnosis to 67% during salvage therapy (55, 56).

The relatively low prevalence of clinically significant weight loss (2.4%, corrected from the erroneous ‘2.6%’ in the prior Methods text) compared with overall nutritional risk (57.3% with PG-SGA-SF ≥ 4) is a notable finding that diverges from some published myeloma and oncology cohorts and warrants explicit mechanistic discussion. At least four factors plausibly account for this discrepancy. First, survivorship bias within the ECOG 0–1 analytic cohort is the most important explanatory factor: patients with the most severe functional and nutritional decline (ECOG ≥2, 38.7% of the screened population) were systematically excluded, and these individuals would disproportionately contribute to acute weight loss prevalence. Second, multiple myeloma has a more indolent disease trajectory compared with rapidly progressive solid tumors—where weight loss of 30–80% is commonly reported (57)—and tends to produce nutritional impairment primarily through symptom-mediated intake reduction and inflammation-driven catabolism rather than through rapid catabolic wasting, a pattern consistent with our PCA findings (PC1 loading on symptom burden rather than weight). Third, unrecorded nutritional support prior to assessment cannot be excluded, as patients actively engaged in treatment at a specialized hematology center with embedded dietitian services may have received informal nutritional guidance attenuating acute weight loss while not preventing symptom-driven risk. Fourth, recall-based weight history is susceptible to systematic underestimation of gradual decline, as patients often normalize slow changes over months. While adenocarcinomas of the pancreas, lung, and gastroesophageal junction typically present with profound weight loss and sarcopenia as dominant features (9, 57), myeloma patients appear to experience nutritional vulnerability primarily through symptom-driven intake impairment, inflammation-mediated hypoalbuminemia, and functional decline rather than acute anthropometric deterioration. This pattern may reflect effective symptom management preventing severe anorexia in some patients, earlier nutritional intervention, or the indolent nature of myeloma compared to rapidly progressive solid tumors (58, 59). Principal component analysis identified distinct vulnerability dimensions, with PC1 (symptom burden/function) and PC2 (inflammation/nutrition) accounting for the largest share of explainable variance. However, the cumulative variance of 38.6% across the first three components suggests that nutritional vulnerability in myeloma is a heterogeneous construct influenced by factors beyond the measured clinical and symptomatic domains (14, 44, 59).

The identification of high symptom burden (ESAS ≥40) as the overwhelmingly dominant independent correlation of nutritional vulnerability (adjusted OR = 5.03.) represents a key finding with important translational implications. This five-fold increased odds exceeded all other correlations including disease stage, treatment line, and inflammation, suggesting that symptom management should be prioritized as a strategy for addressing malnutrition in myeloma populations. Given the cross-sectional design, however, the direction of this association cannot be established with certainty: while high symptom burden plausibly impairs dietary intake and accelerates nutritional decline, reverse causation—wherein malnutrition exacerbates fatigue, pain, and anorexia—is equally plausible and likely bidirectional. Prospective longitudinal studies with repeated assessments are required to evaluate temporality and to determine whether symptom control or nutritional rehabilitation should be the primary intervention target. This finding extends prior work by Ramsenthaler et al. demonstrating strong correlations between symptom burden and nutritional status in palliative cancer populations (60), and aligns with mechanistic studies showing that pro-inflammatory cytokines (IL-6, TNF-α) drive both symptom expression (fatigue, anorexia, pain sensitization) and metabolic derangements (muscle protein catabolism, altered substrate metabolism) characteristic of cancer cachexia (61, 62).

The specific symptom-nutrition relationships observed provide mechanistic insight. Appetite loss demonstrated the strongest correlation with weight loss (*r* = −0.54, *p* < 0.001), consistent with its direct role in reducing dietary intake. However, the equally robust associations with fatigue (*r* = 0.44), pain (*r* = −0.48), and nausea (*r* = −0.46) indicate that symptoms impair nutrition through multiple pathways beyond appetite alone: fatigue limits meal preparation capacity and physical activity necessary for maintaining muscle mass; pain interferes with eating mechanics and reduces motivation for food-related activities; gastrointestinal symptoms (nausea, constipation, diarrhea) directly impair absorption and utilization (19, 20). The bidirectional amplification between nutrition and fatigue—wherein nutritional decline exacerbates fatigue severity, which further impairs dietary adequacy—creates a vicious cycle that may explain the progressive nutritional deterioration observed across treatment phases despite stable disease in many remission-phase patients (22, 63).

The moderate correlation between PG-SGA and ESAS (*r* = 0.41) suggests that while symptom burden is a major driver, nutritional vulnerability is not entirely reducible to symptom severity, supporting the multidimensional phenotype revealed by principal component analysis. Inflammation (PC2), as evidenced by elevated CRP and hypoalbuminemia, represents a parallel pathway that may operate independently of patient-reported symptoms through cytokine-mediated metabolic reprogramming, anabolic resistance, and muscle protein degradation (64, 65). The interpretation of albumin as a nutritional marker warrants particular caution in myeloma given its role in the ISS and its sensitivity to systemic inflammation. While the sensitivity analysis excluding albumin confirms that findings are not materially driven by this criterion (60.1% vs. 61.2% prevalence, *κ* = 0.94), a CRP-stratified analysis would ideally clarify what proportion of the albumin–vulnerability association is inflammation-mediated. CRP data were available for only 55.9% of the analytic cohort (160/286 patients), limiting the statistical power of such a subgroup analysis; this is acknowledged as a limitation and a priority for future prospective studies with complete inflammatory biomarker ascertainment and objective body composition measures. This mechanistic complexity underscores the need for multimodal interventions addressing both symptom palliation and anti-inflammatory strategies. The non-significant associations of ISS stage III (OR = 1.19, *p* = 0.614), treatment line ≥2 (OR = 1.42, *p* = 0.258), and relapsed/refractory disease (OR = 1.39, *p* = 0.277) after multivariable adjustment were unexpected given strong univariate associations and clinical intuition that advanced disease should drive malnutrition.

The attenuation of treatment-related associations in the multivariable model suggests that the impact of advanced disease on nutritional status may be captured by the symptom burden variable. This implies that treatment intensity and disease progression likely influence nutritional vulnerability largely through their association with increased symptoms and functional decline, rather than as independent risk factors (66, 67). The treatment-phase gradient observed in univariate analysis but attenuated in multivariable models is consistent with a mediation framework: later treatment lines are associated with higher nutritional vulnerability, but this relationship operates through accumulated symptom burden, treatment toxicity manifestations (neuropathy, fatigue), and repeated disease-related complications rather than through the number of treatment regimens directly damaging nutritional status.

An alternative explanation involves confounding by indication and selection bias. Patients progressing to multiple treatment lines who remain functional enough to complete comprehensive assessment (ECOG 0–1 requirement) may represent a selected robust subgroup with preserved nutritional resilience despite advanced disease, whereas patients with poor performance status (ECOG ≥2, 38.7% of full cohort) who were unable to complete assessment may have concentrated malnutrition-disease associations. This structural zero problem for ECOG ≥2 in the analytic sample limits our ability to detect disease severity effects that may be most pronounced in the most functionally impaired patients who were systematically excluded by study procedures (68–70).

The finding that 61.6% of patients with moderate-to-high nutritional risk had not received dietitian consultation in the preceding 3 months represents a profound care quality gap with immediate policy implications. This prevalence of unmet nutrition support need exceeds that reported in Western healthcare systems; a multi-center European study by Muscaritoli et al. (71) found 42% of malnourished cancer patients had not received nutrition intervention, while a U.S. study by Prado et al. (72) reported 38% unmet need. The higher prevalence in our Chinese setting may reflect limited dietitian workforce availability in oncology, lack of systematic screening and referral protocols, or patient-side barriers (cost, travel burden, low awareness) that are more pronounced in middle-income healthcare contexts (8, 35, 36, 73).

Regular oral nutritional supplement use was reported by 45.1% of patients overall, representing moderate uptake of this evidence-based intervention. However, the persistence of high nutritional vulnerability rates (57.3% with PG-SGA-SF ≥ 4) despite this substantial ONS utilization raises important questions about supplement effectiveness, appropriateness, or integration with comprehensive nutritional care. Several factors may explain the disconnect between ONS use and nutritional outcomes. First, supplement selection may be suboptimal, with patients self-selecting general nutrition products rather than cancer-specific formulations with appropriate caloric density and protein content. Second, the 61.6% prevalence of unmet dietitian consultation needs suggests that ONS use may be occurring without professional guidance on timing, dosing, or integration with dietary intake, potentially limiting efficacy. Third, cost barriers (55.7% cited expense as limiting factor) may constrain supplement quantity or consistency despite reported use. Fourth, symptom burden—the dominant predictor of nutritional vulnerability (OR = 5.03)—may prevent adequate ONS intake even when products are available, as severe nausea, early satiety, or taste alterations can render supplements unpalatable or poorly tolerated. Meta-analyses by Baldwin et al. and Elia et al. have demonstrated that oral nutritional supplements improve weight, muscle mass, and treatment tolerance in malnourished cancer patients (74, 75), but these benefits require adequate intake, appropriate formulation, and integration with symptom management and dietary counseling—components potentially lacking in our population despite reported ONS use (29). The paradox of high ONS utilization (45.1%) coexisting with high vulnerability (57.3%) and unmet dietitian needs (61.6%) suggests ineffective, unguided supplementation. This likely stems from widespread OTC availability and cultural influences promoting self-directed use of inappropriate products (e.g., general protein powders) without professional oversight. Additionally, our frequency-based data may mask inadequate dosing, while severe symptom burden often renders supplements ineffective by preventing intake. Future interventions must therefore move beyond mere availability to prioritize professional guidance, appropriate formulation, and integrated symptom management to ensure therapeutic benefit.

The systematic barriers assessment revealed multiple, layered obstacles operating at financial (cost 55.7%, insurance gaps), communicative (terminology complexity 39%, inconsistent advice 39%), and systemic (appointment difficulty 37.7%) levels. Notably, geographic barriers related to travel time did not emerge as significant obstacles in this urban, single-center cohort, likely reflecting Taiyuan’s concentrated healthcare infrastructure and public transportation accessibility. This multilevel barrier structure aligns with socioecological models of healthcare access, wherein individual-level factors (health literacy, economic resources) interact with provider-level factors (communication quality, care coordination) and system-level factors (service availability, insurance design) to determine utilization patterns (76, 77). The systematic documentation of this layered barrier profile—spanning financial, communicative, logistical, and digital domains simultaneously within a single validated assessment framework—represents a methodological contribution of this study, providing an empirical foundation for designing multicomponent interventions that must address structural obstacles at all socioecological levels rather than targeting isolated barriers in isolation.

The 48.3% prevalence of food insecurity in this myeloma cohort substantially exceeds general Chinese population estimates (14.2% in urban households, 22.3% in rural households per national food security surveillance) (36), and approaches rates observed in vulnerable U.S. cancer populations where prevalence ranges from 32 to 54% depending on socioeconomic composition (31, 78). The steep income gradient (60.8% in <3,000 RMB/month declining to 21.6% in ≥10,000 RMB/month) demonstrates that food insecurity in cancer is fundamentally an economic access problem rather than knowledge deficit, with financial toxicity from treatment costs reducing resources available for food purchasing (79–81). The persistence of food insecurity as an independent predictor of nutritional vulnerability in fully adjusted models (OR = 2.12, 95% CI: 1.39–3.24) after controlling for income, disease severity, and inflammation indicates that food access operates through pathways beyond measured confounders. Previous research suggests these unmeasured mechanisms include: psychological stress and depression from food worry that suppresses appetite; reduced diet quality when limited budgets force substitution of nutrient-dense foods with cheaper energy-dense options; episodic food depletion that disrupts regular meal patterns; and social isolation when patients avoid communal meals due to shame about household food insufficiency (82, 83).

The profound quality of life impairment associated with nutritional vulnerability—with adjusted mean differences of 35.9 points for global health, 40.3 points for physical functioning, and 40.5 points for role functioning between high and low vulnerability groups—represents some of the largest effect sizes observed in myeloma quality of life research. For comparison, a recent meta-analysis by Mols et al. found that progression from complete response to relapsed disease was associated with only 12-point decrements in global QoL (84), while a study by Osborne et al. reported 18-point differences between ISS stage I and III at diagnosis (85). The fact that nutritional vulnerability is associated with 2-3-fold larger QoL decrements than disease progression or stage suggests it may be among the most impactful modifiable determinants of patient-reported outcomes in myeloma care.

The strong negative correlation (*r* = −0.72) between nutritional vulnerability and global QoL indicates that approximately 52% of quality of life variance is explained by nutritional status and its correlates, representing a potentially modifiable therapeutic target. If nutritional interventions can improve PG-SGA scores by even 2–3 points (moving patients from moderate to low vulnerability), the expected QoL gains of 10–15 points would equal or exceed the benefit of achieving partial response versus stable disease in terms of patient-perceived wellbeing (26, 86). This contextualization underscores that aggressive nutritional assessment and intervention should be considered core components of high-quality myeloma care rather than ancillary supportive services, particularly given the expanding treatment landscape where prolonged survival durations make quality of life preservation increasingly salient (87).

Strengths include the comprehensive assessment framework integrating validated nutrition screening, objective anthropometry, functional capacity testing, biochemical markers, patient-reported outcomes, and systematic barriers evaluation—a methodological combination rarely achieved in hematologic malignancy nutrition research. The inclusion of food insecurity assessment and health equity focus addresses critical gaps in the literature, where most myeloma nutrition studies focus exclusively on clinical and biological determinants while ignoring socioeconomic constraints. The prespecified multivariable models with complete covariate adjustment, effect size reporting, and sophisticated analytical approaches (principal component analysis, sequential adjustment models, interaction testing) enhance rigor and internal validity. The culturally adapted instruments validated in Chinese populations and attention to China-specific dietary patterns (gram-based vegetable/fruit recommendations, Traditional Chinese Medicine utilization) strengthen relevance and applicability to the target healthcare context.

Several limitations warrant consideration. First, the cross-sectional design precludes causal inference; all associations—including the dominant correlation between symptom burden and nutritional vulnerability—should be interpreted as statistical correlates rather than causal relationships, as reverse causation whereby malnutrition exacerbates symptoms remains equally plausible. Prospective longitudinal studies with repeated assessments are required to establish temporality.

Second, the requirement for ECOG 0–1 functional status introduced quantifiable selection bias by excluding an estimated 116 patients (38.7% of those screened), who had significantly greater clinical severity—higher ISS stage III (42.2% vs. 22.4%), greater relapsed/refractory disease (68.1% vs. 49.3%), and lower albumin (36.2 vs. 40.0 g/L). A conservative worst-case scenario assuming complete nutritional vulnerability among excluded ECOG ≥2 patients would raise composite vulnerability prevalence from 61.2% to approximately 75–80%. All prevalence estimates should therefore be interpreted as conservative lower bounds applicable to functionally preserved patients only. Third, the single-center urban tertiary setting limits generalizability to rural Chinese patients, approximately 60% of whom live >100 km from tertiary oncology centers and face substantial access barriers absent in this cohort. Multi-center studies incorporating rural sites are needed for nationally representative estimates. Fourth, objective body composition assessment using dual-energy X-ray absorptiometry or bioelectrical impedance analysis was not performed. Sarcopenia may occur independently of PG-SGA risk scores, potentially underestimating the full burden of functional nutritional vulnerability. Fifth, reliance on self-reported weight history, dietary intake, food security, and symptom severity introduces recall and social desirability bias. Gradual weight decline is particularly susceptible to systematic underestimation, likely contributing to the low observed weight-loss prevalence (2.4%). Finally, CRP data were unavailable for 44.1% of patients, limiting inflammation-stratified subgroup analyses; future studies with complete biomarker ascertainment could clarify the extent to which the albumin–vulnerability association is inflammation-mediated versus nutritionally specific.

## 5 Conclusion

This study identifies nutritional vulnerability as a pervasive and underrecognized sproblem in Chinese patients with multiple myeloma, affecting even functionally preserved individuals and intensifying progressively across treatment phases. High symptom burden demonstrated the strongest independent association with nutritional vulnerability—an association that is likely bidirectional and requires longitudinal confirmation. Despite moderate oral nutritional supplement use, high vulnerability persisted in the absence of structured dietitian involvement, indicating that supplementation alone is insufficient to address symptom-associated nutritional risk. The majority of unmet need was attributable to a systemic failure of identification and referral rather than patient reluctance or geographic access alone. Food insecurity was highly prevalent, exhibited marked socioeconomic gradients, and remained independently associated with nutritional vulnerability beyond clinical factors. The magnitude of quality-of-life impairment linked to nutritional vulnerability exceeded that attributable to disease stage or treatment line, positioning nutrition as a critical, modifiable target in myeloma care. These findings support systematic nutritional screening, routine symptom assessment, automatic dietitian referral for moderate-to-high risk patients, and policy measures to reduce structural and financial barriers to equitable supportive care access.

## Data Availability

The raw data supporting the conclusions of this article will be made available by the authors, without undue reservation.
